# An exploratory study to assess the knowledge, attitudes and practices of Lebanese residents towards acrylamide

**DOI:** 10.1371/journal.pone.0300617

**Published:** 2024-04-16

**Authors:** Nour Kalash, Samer Kharroubi, Rouba Ballout, Fatima Saleh

**Affiliations:** 1 Department of Nutrition & Dietetics, Faculty of Health Sciences, Beirut Arab University, Beirut, Lebanon; 2 Department of Nutrition and Food Sciences, Faculty of Agricultural and Food Sciences, American University of Beirut, Beirut, Lebanon; 3 School of Health and Related Research, The University of Sheffield, Sheffield, United Kingdom; 4 Department of Medical Laboratory Technology, Faculty of Health Sciences, Beirut Arab University, Beirut, Lebanon; Maulana Abul Kalam Azad University of Technology West Bengal, INDIA

## Abstract

**Introduction:**

For years, heat treatment has been an essential method for ensuring mature food that meet the desired quality and safety characteristics. However, this process could lead to the formation of harmful compounds such as acrylamide. In this study we aimed to investigate the knowledge, attitudes and practices (KAPs) of the Lebanese population toward the potential risk associated with acrylamide.

**Materials & methods:**

An online survey (n = 598) was conducted among residents in Lebanon aged 18 years and above. The survey was divided into five sections including participants’ sociodemographic characteristics, knowledge, attitude and practice sections, and some questions related to consumer’s preferences.

**Results & discussion:**

The results showed that the majority of the participants had low food safety knowledge regarding acrylamide. Specifically, 82.9% of the consumers had no idea about the chemical, its formation, the foods with a high risk of acrylamide formation and the health risks associated with its exposure. Despite lack of knowledge, good domestic food practices (storage, pre-treatment) were noticed among participants. Moreover, the majority of consumers (> 80%) showed positive attitude towards proper acrylamide labeling. Participants with a bachelor’s degree appeared to have a more positive attitude toward food safety compared to those with no qualifications (*p*<0.001).

**Conclusion:**

Despite the high consumption of acrylamide by the consumers in Lebanon through fried potatoes, bread, and coffee, the majority have no idea about acrylamide’s presence in food, its sources and its adverse health effects. Raising awareness among the public, involving policy makers in addressing the issue of clear labeling and encouraging the adoption of alternative practices to reduce acrylamide are all crucial to protect consumers’ health in Lebanon and promote healthier food consumption habits.

## Introduction

For safety purposes, heating up food eliminates the risk of microbiological contamination, maintains the nutritional properties of the food and preserves its color, taste and texture [1]. During thermal heating, some hazardous substances are unavoidably produced [2]. A substance that has received significant attention in recent years is acrylamide. Acrylamide is a common processing-related pollutant that is widely found in food, especially starchy foods like potatoes, breads, and coffee when treated at high temperatures [3].

Acrylamide, C3H5NO, is a white odorless, crystalline, water-soluble compound, categorized as an extremely hazardous substance in the United States and as an alarming health risk with serious toxicity in the European Union [4]. Acrylamide in food primarily arises through a chemical reaction called Maillard reaction which takes place when foods rich in carbohydrates such as glucose and fructose react with amino acids mainly asparagine under specific conditions such as high temperatures and low moisture content [5]. Therefore, the type of food, cooking temperature, duration of cooking, moisture levels, storage conditions, composition of raw material are all factors that influence the formation of acrylamide [6]. Changes in these parameters can noticeably influence acrylamide content. For example, soaking potatoes in water before frying can have a positive impact on acrylamide levels in fried potatoes since soaking reduce the amount of glucose in potato chips and thus reduces acrylamide levels. Blanching is another option and has an added benefit, not only does it help decrease glucose levels but also decreases asparagine levels in potatoes [7].

Acrylamide (AA) is a strong neurotoxin that impairs male fertility, results in birth defects, and causes cancer in laboratory tested animals [8]. Despite the evidence indicating its carcinogenicity when experimenting on animals, there has been relatively limited epidemiologic studies conducted on occupational and dietary exposure to AA on humans. In the light of the available data, AA is currently categorized as a “probable human carcinogen” by the International Agency for Research on Cancer (IARC) and as “reasonably anticipated to be a human carcinogen” by the US National Toxicology Program (NTP) [9]. Furthermore, the US Environmental Protection Agency (EPA) classifies acrylamide as “likely to be carcinogenic to humans”. In 2015, the European Food Safety Authority (EFSA) released its first full risk assessment of acrylamide in food, where experts came to the conclusion that AA can potentially increases the risk of developing cancer for consumers of all ages and is considered a public health concern [10] In addition to the carcinogenicity potential of acrylamide, EFSA has recently released in 2022, an updated review reporting substantial evidence for the genotoxicity of acrylamide [11]. Given its potential to be carcinogenic and genotoxic, efforts should be made to increase awareness and decrease exposure. For instance, the UK Food Standards Agency (FSA) recommends that the exposure to acrylamide ‘should be reduced to as low as reasonably achievable (ALARA)’ [12].

As far as we know, this is the first study to explore the acrylamide knowledge, attitudes and practices among Lebanese residents. There are very few reports on quantifying the acrylamide content in food and evaluating the risk of acrylamide consumption. However, there are no KAP studies regarding the risks associated with acrylamide despite its high consumption through fried potatoes, bread and coffee.

The objective of the present work was to assess consumer’s awareness of the risks associated with acrylamide. To this end, we assessed (i) the knowledge, attitudes, and practices of the Lebanese population toward acrylamide consumption, (ii) factors associated with KAP levels, and (iii) measures to raise awareness about the risks of acrylamide which will inform the general population and allow them to make informed decisions about their food choices and cooking habits. It would also enable governmental agencies and regulatory bodies to set guidelines and interventions that can be tailored to reduce its formation and exposure.

## Methods

### Data collection and survey instrument

This cross-sectional study examined knowledge, attitudes, and practices toward the risks of acrylamide among Lebanese residents aged 18 and above. It was ethically approved by the Institutional Review Board (IRB) of Beirut Arab University under approval code 2023-H-0147-HS-R-0518. The English survey (S1 Appendix) adapted from a previous work published by Aly (2019), was translated to Arabic then back-translated to English by two independent official translators who were native Arabic speaker to verify the reliability of the survey [13]. An expert committee then reviewed and compared the Arabic and original English versions to identify any discrepancies. After minor adjustments to wording and format, a prefinal Arabic version of the survey was produced. The prefinal survey was then assessed for content validity through a pilot study with 10 Arabic-speaking Lebanese residents who were not part of the main study sample. Based on their feedback, no adjustments were deemed necessary, as participants found the survey clear and understandable. The committee agreed on the final version of the Arabic survey (S2 Appendix).

Data collection took place between 17 May 2023 and 29 July 2023. An online invitation was shared and posted via different social media platforms (WhatsApp groups, and Facebook pages) where participants were invited to the fill in a blinded on-line questionnaire and a consent form. Written consent was obtained from the participants online as they were asked to read the consent form carefully and indicate their consent by ticking a checkbox to indicate their agreement. Once they agreed to take part in the study, participants proceeded to answering the survey which took approximately 8–10 minutes to complete.

The survey consisted of 42 items. The first 2 questions were meant to determine respondents’ eligibility to participate in the study. Only participants residing in Lebanon and aged 18 or above were included for analysis. Furthermore, the survey was divided into several sections. The first section included questions on the participants’ sociodemographic characteristics such as age, gender, educational level, family status, residence area and income. The second section was composed of direct and indirect questions related to participants’ knowledge about acrylamide. The third section focused on the participants’ attitude towards food labelling, specifically the addition of information related to the presence of acrylamide. A section regarding consumer preferences was also added, there were several questions to gather data on cooking end-point preferences and consumption rate of certain foods like potatoes, bread and coffee. The last section included questions on consumer’s domestic food practices such as storage, preparation, pre-treatment and cooking methods.

### Sample size

Based on the sample size calculator (https://cdn.who.int/media/docs/default-source/ncds/ncd-surveillance/steps/sample-size-calculator.xls?sfvrsn=ee1f4ae8_2) provided by the WHO, the study required a minimum of 577 participants to estimate a prevalence of 50% with a 95% confidence interval and a margin of error of 5%. Additionally, a design effect of 1.5 was taken into consideration. To accommodate for a refusal rate of 10%, 634 participants were included in the study.

### Statistical analysis

Responses were rigorously checked for completeness. Only respondents residing in Lebanon and above the age of 18 were included for data analysis. Data (S3 Appendix) were analyzed using the Statistical Package for the Social Sciences (SPSS) version 27.0 (SPSS Inc., Chicago, IL, USA). Descriptive statistics were carried out as frequencies and proportions for categorical variables. Each correct answer received a score of 1 point, while incorrect answers were assigned a score of 0 point. For each participant, the total knowledge and attitude scores were determined by adding the number of correct answers. The total score was dichotomized as having lower or higher level of acrylamide knowledge and attitude. For each variable, the cut-off point was set to be the mean score of correct answers. Participants with KAP scores below or equal to the mean score were classified as having low KAP levels; whereas, those who scored above the mean score were considered to have a high KAP level. The association between two categorical variables was calculated using the chi-square test. Simple logistic regression and multiple logistic regressions were employed to identify factors associated with knowledge and attitude levels. The sociodemographic characteristics represented the independent variables, while total knowledge and attitude score ratings represented the dependent variables. Characteristics that showed statistical significance in the simple analysis were included as independent variables in the multiple models. Results from the logistic regression analyses were expressed as odds ratios (OR) with 95% CI. For all analyses, a *p*-value of less than 0.05 was considered statistically significant.

## Results

### Sociodemographic characteristics of participants

The total number of participants was 634, of which 36 participants were disqualified. Disqualification criteria such as ‘do not live in Lebanon’ (n = 21) and ‘under 18 years old’ (n = 15), brought the final qualified participants to 598. Table 1 presents the sociodemographic characteristics of the study population. Results showed that more than half of the participants were females (61.2%) and above the age of 34 (62%). The study sample included consumers from all over Lebanon, mostly from the city (92.8%). More than one half of the participants held a university degree (58.3%). Approximately 19% of the participants reported a monthly income exceeding 500$. Furthermore, 61% of the respondents were married. Unsurprisingly, the majority of consumers (48.1%) rely on the internet and social media as the primary source of information.

**Table 1:** Sociodemographic characteristics among participants.

Sociodemographic characteristics		n (%)
Gender	Male	232 (38.8)
	Female	366 (61.2)
Age	18–24	80 (13.4)
	25–34	165 (27.6)
	35–44	180 (30.1)
	45–54	110 (18.4)
	55–64	51 (8.5)
	65+	12 (2)
Area of residency	City/ Town	555 (92.8)
	Countryside	43 (7.2)
Education level	No qualifications	53 (8.9)
	Secondary school	196 (32.8)
	Bachelor degree	267 (44.6)
	Master/ Doctorate degree	82 (13.7)
Monthly income	<100$	179 (29.9)
	100–500$	307 (51.3)
	≥500$	112 (18.7)
Family status	Single	204 (34.1)
	Married without children	60 (10)
	Married with children	305 (51)
	Other	29 (4.8)
Source of information	School/ College	25 (19.1)
	Internet/ Social media	63 (48.1)
	Family/ Friends	20 (15.3)
	TV	7 (5.3)
	Other (food safety authority, journal/magazine, several sources)	16 (12.3)

### Participant’s knowledge

Tables 2 and 3. summarizes acrylamide knowledge of the consumers. The overall mean score for knowledge was 2.22 ±0.601, which highlights a low level. The majority of the consumers had no idea about acrylamide in food (82.9%), and what are the foods that have the potential to contribute to high acrylamide exposure (83.8%). Furthermore, the majority did not know the conditions under which acrylamide might be formed in food (91.6%), and what are the negative health effects from this harmful compound (95.5%). When asked about the reason for soaking and not soaking raw potatoes, and why they parboil potatoes before cooking, more than half of the respondents gave valid answers (52.6%, 59.6%).

**Table 2:** Summary of some knowledge questions (direct).

	Question	Good knowledge n (%)	Bad knowledge n (%)
1.	Have you previously heard about acrylamide in food	102 (17.1)	496 (82.9)
2.	Which of the following foods have the potential to contribute to high acrylamide exposure?	97 (16.2)	501 (83.8)
3.	Why do you think so?	61 (10.2)	537 (89.8)
4.	Under what conditions do you think acrylamide might be formed in food?	50 (8.4)	548 (91.6)
5.	What do you think are the negative health effects from this harmful compound?	27 (4.5)	571 (95.5)
	Total mean of correct answer: 2.22 ±0.601		

**Table 3:** Summary of some knowledge questions (indirect).

	Question	Right answer n (%)	Wrong answer n (%)
1.	For what reason do you store raw potatoes there?	128 (22.4)	444 (77.6)
2.	For what reason do you soak or not soak raw potatoes?	209 (52.6)	189 (47.5)
3.	Why do you parboil the potatoes before cooking?	56 (59.6)	38 (40.4)

### Participant’s attitude

Table 4. summarizes consumers’ attitude towards acrylamide labeling. Consumers showed positive attitude towards proper acrylamide labeling; with a total mean score of 2.46 ±0.869. The majority of the respondents agreed that food packaging should contain information regarding the content and safe levels of acrylamide (86.8%) as well as about the potential formation of acrylamide in the product if the cooking instructions are not properly followed (90.6%). More than two thirds of the participants would purchase the food product if its label indicated that acrylamide was present at a safe level (68.9%).

**Table 4:** Consumers’ attitude towards acrylamide labeling.

	Question	Positive attitude n (%)	Negative attitude n (%)
1.	Should food packaging contain information about the content and safe level of acrylamide?	519 (86.8)	79 (13.2)
2.	Should food packaging contain information about the potential formation of acrylamide in the product if the cooking instructions are not properly followed?	542 (90.6)	56 (9.4)
3.	If a product’s label listed acrylamide at a safe level, would you buy the product?	412 (68.9)	186 (31.1)
	Total mean of correct answer: 2.46 ±0.869		

### Participants’ consumption rates and personal preferences

Table 5. summarizes how often consumers consume some food products. More than half of the respondents eat chips or roasted potatoes more than twice per week (62.4%), and drink at least 2 cup of coffee per day (62.2%). Most of them eat 1–2 slices of bread per day (72.7%). Table 6 summarizes consumer’s preferences when it comes to food consumption. More than half of them usually buy white bread (60.4%) and almost half of the respondents drink roasted ground coffee (49%).

**Table 5:** Summary of some consumer’s consumption rate of different products.

Consumption rates		n (%)
How often do you eat chips or roasted potatoes?	Never	6 (1)
	Once per month	69 (11.5)
	Once per week	150 (25.1)
	2–3 times per week	290 (48.5)
	Daily	83 (13.9)
How many slices of bread do you usually eat per day?	Rarely	65 (10.9)
	Half slice	2 (0.3)
	1–2	435 (72.7)
	3–5	78 (13)
	>5	12 (2)
	Other	1 (0.2)
	I do not eat bread	5 (0.8)
How many cups of coffee do you drink daily	1	128 (21.4)
	2	193 (32.3)
	3	98 (16.4)
	4	37 (6.2)
	5	24 (4)
	>5	20 (3.3)
	I don’t drink coffee	98 (16.4)

**Table 6:** Summary about consumer’s preferences.

Consumer’s preferences	n (%)
White	361 (60.4)
Brown	100 (16.7)
Whole wheat	52 (8.7)
Oat	4 (0.7)
Bra	4 (0.7)
Combination of white, brown and whole wheat	62 (10.4)
I do not use bread	15 (2.5)
Instant coffee	188 (31.4)
Roasted ground coffee	293 (49)
Coffee substitutes	27 (4.5)
Other (tea, Turkish…)	6 (1.1)
I don’t drink coffee	84 (14)

### Participant’s practices

Table 7. summarizes domestic food practices that could negatively affect the safety of the product. Good practices were noticed among participants. The majority of the respondents store raw potatoes in appropriate places such as the pantry away from sunlight (90.8%). They also wash the potatoes after peeling which reduces the risk of acrylamide formation (93.6%). Bad practices were also observed. Only 9.2% of the participants abided to the instructed time label when cooking pre-cooked food. And only 21.8% applied safe cooking methods.

**Table 7:** Summary of some practice questions.

	Question	Good practice n (%)	Bad practice n (%)
1.	Where do you usually store raw potatoes	543 (90.8)	55 (9.2)
2.	Do you usually wash the potatoes after peeling	512 (93.6)	35 (6.4)
3.	Do you soak peeled or cut potatoes before frying or roasting?	317 (56.7)	242 (43.3)
4.	Do you parboil the potatoes before cooking?	48 (8.7)	506 (91.3)
5.	When you cook pre-cooked food how do you estimate cooking time?	54 (9.2)	532 (90.8)
6.	What heat setting on your oven do you select to roast fresh potatoes?	182 (30.6)	412 (69.4)
7.	How do you normally cook chips?	127 (21.8)	456 (78.2)

### Association between the KAP scores and demographic characteristics

The Chi-square analysis as summarized in Tables 8 and 9 showed the association between the sociodemographic characteristics and the level of knowledge and attitude towards acrylamide, respectively. There was significant association between respondents’ knowledge levels of acrylamide and 3 demographic variables. Gender (*p* = 0.042), residency (*p* = 0.036), and education level (*p* = 0.032) were found to affect the level of knowledge.

**Table 8:** Effect of sociodemographic characteristics on participants’ knowledge.

	Total (n = 598)	Insufficient Knowledge n(%)	Good Knowledge n(%)	Significance
**Gender**				
Male	232	204 (87.9)	28 (12.1)	***P* = 0.042**
Female	366	299 (81.7)	67 (18.3)	*X*^*2*^ = 4.134
**Age**				
18–24	80	75 (93.8)	5 (6.3)	*P* = 0.149
25–34	165	133 (80.6)	32 (19.4)	*X*^*2*^ = 8.131
35–44	180	149 (82.8)	31 (17.2)	
45–54	110	91 (82.7)	19 (17.3)	
55–64	51	45 (88.2)	6 (11.8)	
65+	12	10 (83.3)	2 (16.7)	
**Residency**				
City/ Town	555	462 (83.2)	93 (16.8)	***P* = 0.036**
Countryside	43	41 (95.3)	2 (4.7)	*X*^*2*^ = 4.377
**Education level**				
No qualifications	53	49 (92.5)	4 (7.5)	***P* = 0.032**
Secondary school	196	173 (88.3)	23 (11.7)	*X*^*2*^ = 8.792
Bachelor degree	267	216 (80.9)	51 (19.1)	
Mater/Doctorate degree	82	65 (79.3)	17 (20.7)	
**Monthly income**				
<100$	179	157 (87.7)	22 (12.3)	*P* = 0.074
100–500$	307	259 (84.4)	48 (15.6)	*X*^*2*^ = 5.217
≥500$	112	87 (77.7)	25 (22.3)	
**Family status**				
Single	204	178 (87.3)	26 (12.7)	*P* = 0.212
Married without children	60	46 (76.7)	14 (23.3)	*X*^*2*^ = 4.505
Married with children	305	256 (83.9)	49 (16.1)	
Other	29	23 (79.3)	6 (20.7)	

*A score >2.22 was considered as good knowledge

**Table 9:** Effect of sociodemographic characteristics on participants’ attitude.

	Total (n = 598)	Negative attitude n(%)	Positive attitude n(%)	Significance
**Gender**				
Male	232	59 (25.4)	173 (74.6)	***p <0*.*001***
Female	366	151 (41.3)	314 (58.7)	*X*^*2*^ = 15.608
**Age**				
18–24	80	43 (53.8)	37 (46.3)	***p* = 0.009**
25–34	165	58 (35.2)	107 (64.8)	*X*^*2*^ = 15.339
35–44	180	53 (29.4)	127 (70.6)	
45–54	110	36 (32.7)	74 (67.3)	
55–64	51	16 (31.4)	35 (68.6)	
65+	12	4 (33.3)	8 (66.7)	
**Residency**				
City/ Town	555	189 (34.1)	366 (65.9)	***p* = 0.05**
Countryside	43	21 (48.8)	22 (51.2)	*X*^*2*^ = 3.828
**Education level**				
No qualifications	53	24 (45.3)	29 (54.7)	***p* = 0.003**
Secondary school	196	69 (35.2)	127 (64.8)	*X*^*2*^ = 13.742
Bachelor degree	267	77 (28.8)	190 (71.2)	
Mater/Doctorate degree	82	40 (48.8)	42 (51.2)	
**Monthly income**				
<100$	179	41 (22.9)	138 (77.1)	***p* <0.001**
100–500$	307	121 (39.4)	186 (60.6)	*X*^*2*^ = 17.148
≥500$	112	48 (42.9)	64 (57.1)	
**Family status**				
Single	204	77 (37.7)	127 (62.3)	***p* = 0.017**
Married without children	60	16 (26.7)	44 (73.3)	*X*^*2*^ = 10.257
Married with children	305	100 (32.8)	205 (67.2)	
Other	29	17 (58.6)	12 (41.4)	

*A score >2.46 was considered as positive attitude

Moreover, results revealed significant differences (*p* <0.05) in the attitude section with the gender, age, residency, educational level, monthly income and family status as shown in Table 9.

### Simple and multiple logistic regression analyses

Simple logistic regression showed that five predictors were significantly associated with participants’ knowledge scores (Table 10), including 1- gender (OR = 1.633, *p* = 0.043), where female participants were more likely to have better knowledge than male, 2- age (25–34: OR = 3.609, *p* = 0.011; 35–44: OR = 3.121, *p* = 0.023; *45–54*: OR = *3*.*132*, *p = 0*.*03*), 3- education level (Bachelor’s: OR = 2.892, *p* = 0.05; Master’s/ Doctorate Degree: OR = 3.204, *p* = 0.047), 4- monthly income (OR = 2.051, *p* = 0.025), and 5- family status (OR = *2*.*084*, *p = 0*.*048*). Multiple regression analyses showed that only age (25–34: *OR* = 3.756, *p* = 0.012; 35–44: OR = 3.249, *p* = 0.032; 45–54: OR = *3*.*169*, *p = 0*.*048*) was significantly associated with knowledge score.

**Table 10:** Simple and multiple logistic regression analysis for the associations of the socio-demographic characteristics with the likelihood of having good knowledge.

	Simple logistic regression	Multiple logistic regression
	OR 95%CI	OR 95%CI
**Gender**		
Male	1	1
Female	**1.633(1.015,2.627), *p* = 0.043**	1.598(0.974,2.622), *p* = 0.64
**Age**		
18–24	1	1
25–34	**3.609(1.349,9.656), *p* = 0.011**	**3.756(1.337,10.548), *p* = 0.012**
35–44	**3.121(1.166,8.353), *p* = 0.023**	**3.249(1.105,9.556), *p* = 0.032**
45–54	**3.132(1.116,8.785), *p* = 0.03**	**3.169(1.012,9.927), *p* = 0.048**
55–64	2(0.577,6.932), *p* = 0.274	2.307(0.593,8.977), *p* = 0.228
65+	3(0.512,17.57), *p* = 0.223	3.861(0.593,25.125), *p* = 0.157
**Residency**		
City/ Town	1	
Countryside	0.242 (0.058,1.019), *p* = 0.053	
**Education level**		
No qualifications	1	1
Secondary school	1.629(0.538,4.933), *p* = 0.388	1.953(0.629,6.063), *p* = 0.247
Bachelor degree	**2.892(0.998,8.381), *p* = 0.05**	2.945(0.976,8.881), *p* = 0.055
Mater/Doctorate degree	**3.204(1.014,10.124), *p* = 0.047**	2.77(0.843,9.153), *p* = 0.093
**Monthly income**		
**<**100$	1	1
100–500$	1.323(0.769,2.274), *p* = 0.312	1.084(0.609,1.93), *p* = 0.784
≥500$	**2.051(1.092,3.850), *p* = 0.025**	1.655(0.843,3.251), *p* = 0.143
**Family status**		
Single	1	1
Married without children	**2.084(1.008,4.307), *p* = 0.048**	1.753(0.781,3.935), *p* = 0.174
Married with children	1.310(0.785,2.188), *p* = 0.301	0.938(0.518,1.699), *p* = 0.833
Other	1.786(0.665,4.798), *p* = 0.25	1.547(0.541,4.424), *p* = 0.416

Simple logistic regression analysis also showed that the variables significantly associated with the likelihood to have positive attitude in the study population included the gender (OR = 0.486, *p* < 0.001), age (25–35: OR = 2.144, p = 0.006; 35–44: OR = 2.789, *p <0*.*001*; 45–54: OR = 2.389, p = 0.004; 55–64: OR = 2.542, p = 0.013), residence (OR = 0.541, *p* = 0.05), educational level (Bachelor’s: OR = 3.619, *p* <0.001; Master’s/ Doctorate Degree: OR = 2.042, *p* = 0.02), family status (Other: OR = 0.428, p = 0.036), and monthly income (100–500$: OR = 0.457, *p <0*.*001; 500$+*: *OR = 0*.*396*, *p <0*.*001)*. (Table 11). The results of the multiple logistic analysis showed that all of the mentioned variables remained significant except for the residency. 1-gender (OR = 0.494, *p <0*.*001)*, 2-age (25–35: OR = 2.266, p = 0.01; 35–44: OR = 2.876, *p = 0*.*002*; 45–54: OR = 2.458, p = 0.016; 55–64: OR = 2.664, p = 0.028), 3-educational level (Bachelor’s: OR = 3.256, *p <0*.*001*), 4-family status (Other: OR = 0.388, *p* = 0.036), and 5-monthly income (100–500$: OR = 0.366, *p <0*.*001; 500$+*: *OR = 0*.*318*, *p <0*.*001)*.

**Table 11:** Simple and multiple logistic regression analysis for the associations of the socio-demographic characteristics with the likelihood of having positive attitude.

	Simple logistic regression	Multiple logistic regression
	OR 95%CI	OR 95%CI
**Gender**		
Male	1	1
Female	**0.486(0.338,0.697), *p*<0.001**	**0.494(0.333,0.732), *p*<0.001**
**Age**		
18–24	1	1
25–34	**2,144(1.245,3.692), *p* = 0.006**	**2.266(1.22,4.208), *p* = 0.01**
35–44	**2.785(1.616,4.798), *p*<0.001**	**2.876(1.479,5.595), *p* = 0.002**
45–54	**2.389(1.32,4.323), *p* = 0.004**	**2.458(1.183,5.108), *p* = 0.016**
55–64	**2.542(1.217,5.312), *p* = 0.013**	**2.664(1.111,6.398), *p* = 0.028**
65+	2.324(0.647,8.344), *p* = 0.196	1.834(0.459,7.326), *p* = 0.39
**Residency**		
City/ Town	1	1
Countryside	**0.541 (0.29,1.009), *p* = 0.05**	0.657 (0.336,1.287)), *p* = 0.221
**Education level**		
No qualifications	1	1
Secondary school	1.523(0.823,2.818), *p* = 0.18	1.8(0.919,3.524), *p* = 0.087
Bachelor degree	**2.042(1.18,3.729), *p* = 0.02**	**3.256(1.658,6.394), *p*<0.001**
Mater/Doctorate degree	0.869(0.435,1.737), *p* = 0.691	1.448(0.678,3.095), *p* = 0.339
**Monthly income**		
**<**100$	1	1
100–500$	**0.457(0.301,0.693), *p*<0.001**	**0.366 (0.231,0.581), *p*<0.001**
≥500$	**0.396(0.396,0.661), *p*<0.001**	**0.318 (0.179,0.562), *p*<0.001**
**Family status**		
Single	1	1
Married without children	1.667(0.881,3.157), *p* = 0.117	1.394(0.676,2.873), p = 0.368
Married with children	1.243(0.858,1.8), *p* = 0.25	0.908(0.563,1.464), p = 0.692
Other	**0.428(0.194,0.944), *p* = 0.036**	**0.388 (0.16, 0.938), p = 0.036**

## Discussion

Acrylamide is a heat-induced food contaminant produced during various cooking methods such as baking, frying, grilling, toasting or roasting [4]. While eliminating acrylamide from our diet is unfeasible, controlling its formation and reducing its intake is possible and essential, particularly in developing countries where exposure to AA is high and awareness of its health risks remains low [14].

In Lebanon, the average consumption of acrylamide from potato and corn chips among the population (age 3–75 years) was found to be 7–24 times higher than the intake of 0.14 μg/kg-bw/day as estimated by the WHO [15]. Additionally, Lebanese have a strong affinity for coffee and caffeinated beverages. A study by El-Zakhem Naous et al (2018), revealed that daily consumption of acrylamide from Lebanese coffee and Espresso was found to be higher than the risk intake for carcinogenicity and neurotoxicity set by the WHO [16]. In light of these alarming findings, it is crucial to assess people’s understanding of acrylamide, its presence in food, their attitudes toward its potential health effects, their consumption rate and their domestic practices regarding storage, pre-treatment and cooking. By addressing these gaps, informed strategies can be implemented to educate the public about acrylamide and encourage healthier cooking and dietary practices to minimize potential risks.

Our study was the first in Lebanon to monitor the level of knowledge, attitudes and practices regarding acrylamide among residents. The main sources of acrylamide in the diet of Lebanese consumers are potatoes (including fries, chips, and baked products), bread, and coffee. Therefore, our study focused on these items, examining their intake, frequency, preferred types and reasons behind their choices. Alarmingly, the majority of Lebanese adult consumers (82.9%) have no idea about acrylamide or the foods that have the potential to contribute to high acrylamide exposure (83.8%). Furthermore, the survey findings revealed that a significant majority of respondents were unaware of the conditions under which acrylamide is formed in food (91.6%) and the potential negative health effects associated with this compound (95.5%). These results emphasize the need for awareness campaigns. This was similar to a study conducted among Polish medical school students which showed that the majority of the studied population (93%) had not heard about acrylamide before, and none had knowledge of its occurrence and formation [17].

However, when asked about the reason for soaking and not soaking raw potatoes, and why they parboil potatoes before cooking, more than half of the respondents gave valid answers. Residency was shown to affect knowledge level. Individuals residing in urban areas had higher levels of knowledge regarding acrylamide than those residing in rural areas. Understanding these rural disparities can help tailor educational campaigns effectively. Most of the participants showed a promising attitude toward labeling food packages with acrylamide levels and safe intake (>80%). This could indicate the willingness among the public to gain knowledge about acrylamide in food. Moreover, participants with a bachelor’s degree were more likely to show a positive attitude toward acrylamide labeling compared to those with no qualifications (p<0.001). This was in accordance with another study by Kowalska et al. where over 50% of respondents thought it was essential that packages contain information about even the smallest quantity of a harmful compound. In our study, more than 60% of respondents reported consuming chips or roasted potatoes more than twice per week, at least 2 cup of coffee per day and 1–2 slices of bread per day. Coffee consumption was higher than in a Polish study conducted by Kowalska et al. [17] where 49% had 2–3 cups daily. According to Bekas and colleagues, people who drink coffee on a daily basis tend to have a daily intake of acrylamide around 3.1 μg. However, people who consume coffee a few times weekly have an average daily intake of acrylamide of around 1.4 μg [18]. A study by Ahrneá et al. showed that white bread baked at 260 degrees for 15 minutes has the highest amount of acrylamide [19]. Furthermore, the majority of our participants favored fried or roasted potatoes with intense crust color, referring to them as crunchier and tastier, leaving them at high risk of acrylamide exposure.

Surprisingly, most of the participants showed good practices that help reduce acrylamide formation. For instance, the majority (90.8%) store potatoes in appropriate conditions like pantry, away from sunlight, giving that storage conditions strongly impact the level of reducing sugars in potatoes [20]. Moreover, 93.6% of the participants wash potatoes after peeling, this helps reduce the quantity of starch. However, only 56.7% soak potatoes in water before frying or roasting. A study by Kita et al. showed the importance of soaking and blanching potatoes before cooking. They deduced that soaking decreases the content of reducing sugars and consequently acrylamide formation; and that the most efficient method was blanching potatoes for 3 min at 70 degrees [21]. This finding highlights an interesting aspect of the participants’ behavior. While the majority of the respondents have limited understanding of acrylamide and its potential negative health effects, their cultural practices or culinary traditions may have led them to adopt certain practices that inadvertently reduce acrylamide formation. This demonstrates the influence of cultural or social factors on individuals’ practices, even in the absence of explicit knowledge.

All in all, this study revealed that a significant proportion of Lebanese residents consume foods such as bread, coffee and potatoes that are highly susceptible to acrylamide formation due to their preparation methods. Alarmingly, they lack knowledge about this harmful compound, its sources and its negative health effects. Therefore, it is crucial to raise awareness campaigns targeting schools, hospitals, and community centers.

Our observations may have been impacted by several limitations. For instance, online surveys may result in selection bias since some groups in a population may not have equal access to the internet and social media platforms. The latter may comprise elderly, rural communities, or unprivileged population. Despite limitations, our study can be used to justify the development of direct-to-consumer interventions that targets harmful compounds in food such as acrylamide.

In conclusion, Lebanese consumers had positive attitude toward acrylamide labeling. However, they had gaps in knowledge and some practices related to acrylamide; mainly its formation in food, its negative health impact, and safe cooking methods. Therefore, it is important to share the results of this study with relevant policymakers, such as health ministries, food regulatory agencies, and consumer protection agencies and to assist in the development of regulations and guidelines for adding information regarding acrylamide content on food packages so that consumers can make informed choices. Furthermore, encouraging the food industry to adopt practices that minimize acrylamide formation is essential, thereby raising awareness among the public, involving policy makers in addressing the issue of clear labeling and encouraging the adoption of alternative practices to reduce acrylamide are all crucial to protect consumers’ health in Lebanon and promote healthier food consumption habits.

## Supporting information

S1 Appendix Knowledge, attitudes and practices regarding acrylamide in food among the Lebanese residents (English version). (DOCX)

S2 Appendix Knowledge, attitudes and practices regarding acrylamide in food among the Lebanese residents (Arabic version). (DOCX)

S3 Appendix Data of the study. (XLSX)
