# Supplementary material for: An exploratory study to assess the knowledge, attitudes and practices of Lebanese residents towards acrylamide
Source: PLoS One. 2024 Apr 16;19(4):e0300617. doi: 10.1371/journal.pone.0300617 (PMC11020536; doi:10.1371/journal.pone.0300617)
Supplement: S1 Appendix — (DOCX) [file pone.0300617.s001.docx]

**Survey about the Knowledge, Attitudes and Practices regarding Acrylamide in food among the Lebanese residents**

| 1. **Residence** | | | | | | | | | | |
| --- | --- | --- | --- | --- | --- | --- | --- | --- | --- | --- |
|  | | | | | | | | | | |
|  | I live in Lebanon | | | | | | | | | |
|  | | | | | | | | | | |
|  | I do not live in Lebanon | | | | | | | | | |
|  | | | | | | | | | | |
| 1. **Is your age over 18 years old?** | | | | | | | | | | |
|  | | | | | | | | | | |
|  | Yes | | | | | | | | | |
|  | | | | | | | | | | |
|  | No | | | | | | | | | |
|  | | | | | | | | | | |
| \| 1. **What is your gender?** \| \| \| \| --- \| --- \| --- \| \|  \| \| \| \|  \| Male \| \| \|  \| \| \| \|  \| Female \| \| \| 1. **Family status** \| \| \| \|  \| \| \| \| Single \| \| \|  \| \| \| \| Married with children \| \| \|  \| \| \| \| Married without children \| \| \|  \| \| \| \| Other \| \| \| 1. **What is the highest level of education you have completed?** \| \| \| \|  \| \| \| \|  \| Bachelor’s Degree \| \| \|  \| \| \| \|  \| Secondary School \| \| \|  \| \| \| \|  \| Master/ Doctorate Degree \| \| \|  \| \| \| \|  \| No qualifications \| \| \| 1. **Average household Income is** \| \| \| \|  \| \| \| \|  \| Less than 100$ \| \| \|  \| \| \| \|  \| Between 100 $ and 500 $ \| \| \|  \| \| \| \|  \| More than 500 $ \| \| \|  \| \| \| \| \| 1. **What is your age range?** \| \| --- \| \| \| \| \|  \| 18-24 \| \| \|  \| \| \| \|  \| 25-34 \| \| \|  \| \| \| \|  \| 35-44 \| \| \|  \| \| \| \|  \| 45-54 \| \| \|  \| \| \| \|  \| 55-64 \| \| \|  \| \| \| \|  \| 65+ \| \| | | | | | | | | | | |
|  | | | | | | | | | | |
|  | | | | | | | | | | |
| \| 1. **What is your residence area?** \| \| \| \| --- \| --- \| --- \| \|  \| \| \| \|  \| \| City/Town \| \|  \| \| \| \|  \| \| Countryside \| \|  \| \| \| \| 1. **Have you previously heard about ACRYLAMIDE in food?** \| \| \| \|  \| \| \| \|  \| Yes \| \| \|  \| \| \| \|  \| No (skip to question № 7) \| \|  1. **Which of the following foods have the potential to contribute to high ACRYLAMIDE exposure? (Tick as many as are relevant).** | | | | | | | | | | |
|  | | | | | | | | | | |
|  | Green vegetables | | | | | | | | | |
|  | | | | | | | | | | |
|  | Fruit | | | | | | | | | |
|  | | | | | | | | | | |
|  | Milk | | | | | | | | | |
|  | | | | | | | | | | |
|  | Meat | | | | | | | | | |
|  | | | | | | | | | | |
|  | Fish | | | | | | | | | |
|  | | | | | | | | | | |
|  | Bread | | | | | | | | | |
|  | | | | | | | | | | |
|  | Eggs | | | | | | | | | |
|  | | | | | | | | | | |
|  | Potatoes | | | | | | | | | |
|  | | | | | | | | | | |
|  | Poultry | | | | | | | | | |
|  | | | | | | | | | | |
|  | Coffee | | | | | | | | | |
|  | | | | | | | | | | |
|  | Biscuits / Crackers / Cereals | | | | | | | | | |
|  | | | | | | | | | | |
|  | Cereals | | | | | | | | | |
|  | | | | | | | | | | |
| 1. **Why do you think so?** | | | | | | | | | | |
|  | | | | | | | | | | |
|  | I do not know | | | | | | | | | |
|  | | | | | | | | | | |
|  | I choose those food because: (Please specified in the below box) | | | | | | | | | |
|  | | | | | | | | | | |
| I think: | | | | | | | | | | |
| \|  \| \| --- \| | | | | | | | | | | |
|  | | | | | | | | | | |
| 1. **Under what conditions do you think acrylamide might be formed in foods? (Tick as many as are relevant).** | | | | | | | | | | |
|  | | | | | | | | | | |
|  | During freezing | | | | | | | | | |
|  | | | | | | | | | | |
|  | During preparation (e.g. peeling, cutting, etc.). | | | | | | | | | |
|  | | | | | | | | | | |
|  | In a refrigerator. | | | | | | | | | |
|  | | | | | | | | | | |
|  | During storing in room temperature. | | | | | | | | | |
|  | | | | | | | | | | |
|  | High temperature cooking (such as frying, roasting, or baking). | | | | | | | | | |
|  | | | | | | | | | | |
|  | After eating/ digestion | | | | | | | | | |
|  | | | | | | | | | | |
|  | I do not know. | | | | | | | | | |
|  | | | | | | | | | | |
| 1. **Where do you usually store raw potatoes?** | | | | | | | | | | |
|  | | | | | | | | | | |
|  | Fridge | | | | | | | | | |
|  | | | | | | | | | | |
|  | Freezer | | | | | | | | | |
|  | | | | | | | | | | |
|  | In a closet or a pantry at room temperature. | | | | | | | | | |
|  | | | | | | | | | | |
|  | I do not use raw potatoes at all (skip to question № 8) | | | | | | | | | |
|  | | | | | | | | | | |
|  | Other (Please specify) | | | | | | | | | |
| \|  \| \| --- \| | | | | | | | | | | |
|  | | | | | | | | | | |
| 1. **For what reason do you store raw potatoes there?** | | | | | | | | | | |
| \|  \| \| --- \| | | | | | | | | | | |
|  | | | | | | | | | | |
| 1. **For how long would you say you usually store raw potatoes from date of purchase?** | | | | | | | | | | |
|  | | | | | | | | | | |
|  | 0-3 days | | | | | | | | | |
|  | | | | | | | | | | |
|  | 4-6 days | | | | | | | | | |
|  | | | | | | | | | | |
|  | 7-9 days | | | | | | | | | |
|  | | | | | | | | | | |
|  | For 2 weeks | | | | | | | | | |
|  | | | | | | | | | | |
|  | More than 2 weeks | | | | | | | | | |
|  | | | | | | | | | | |
|  | I cannot remember. | | | | | | | | | |
|  | | | | | | | | | | |
| 1. **Do you usually peel the potatoes before cooking?** | | | | | | | | | | |
|  | | | | | | | | | | |
|  | Always | | | | | | | | | |
|  | | | | | | | | | | |
|  | Never (skip to question № 12) | | | | | | | | | |
|  | | | | | | | | | | |
|  | Usually | | | | | | | | | |
|  | | | | | | | | | | |
|  | Rarely | | | | | | | | | |
|  | | | | | | | | | | |
|  | Not sure | | | | | | | | | |
|  | | | | | | | | | | |
| 1. **Do you usually wash the potatoes after peeling?** | | | | | | | | | | |
|  | | | | | | | | | | |
|  | Always | | | | | | | | | |
|  | | | | | | | | | | |
|  | Never | | | | | | | | | |
|  | | | | | | | | | | |
|  | Usually | | | | | | | | | |
|  | | | | | | | | | | |
|  | Rarely | | | | | | | | | |
|  | | | | | | | | | | |
|  | Not sure | | | | | | | | | |
|  | | | | | | | | | | |
| 1. **Which size do you usually cut potatoes for roasting? (Tick as many as are relevant)** | | | | | | | | | | |
|  | | | | | | | | | | |
|  | Whole | | | | | |  | | Medium size cubes | |
| 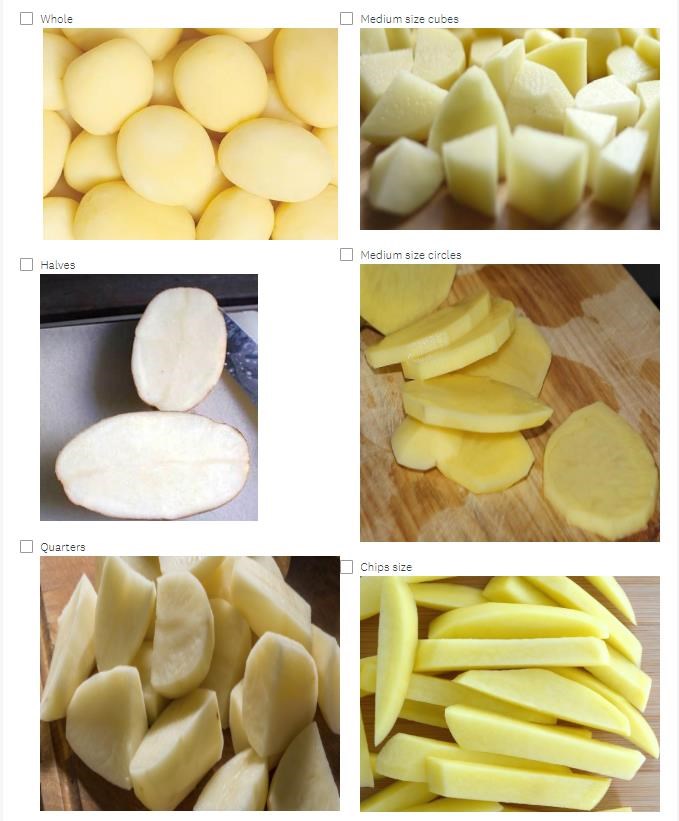 | | | | | | |  | | | |
|  | | | | | | | | | | |
|  | Halves | | | | | |  | | Medium size circles | |
| 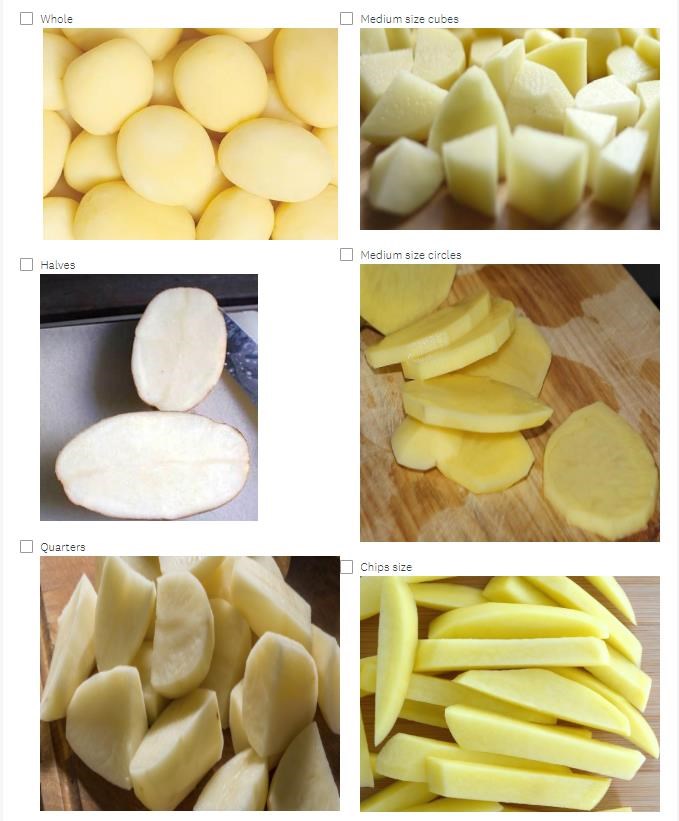 | | | | | | | 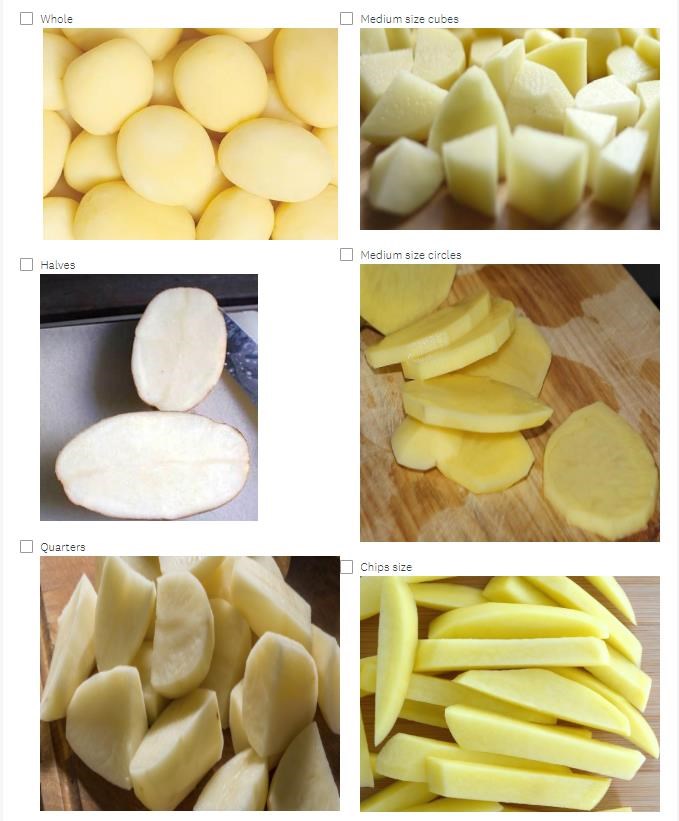 | | | |
|  | | | | | | | | | | |
|  | Quarters | | | | | |  | | Chips size | |
| 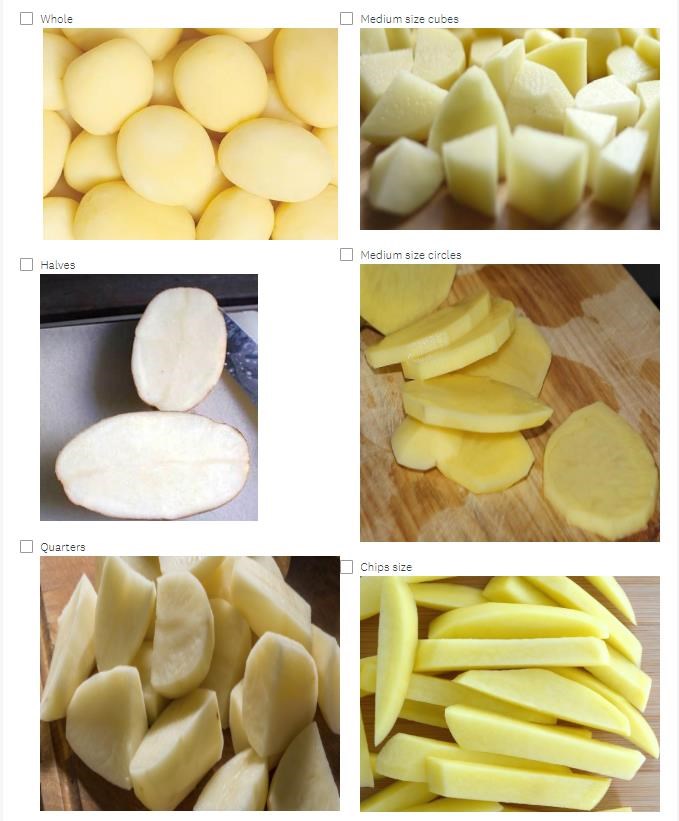 | | | | | | | 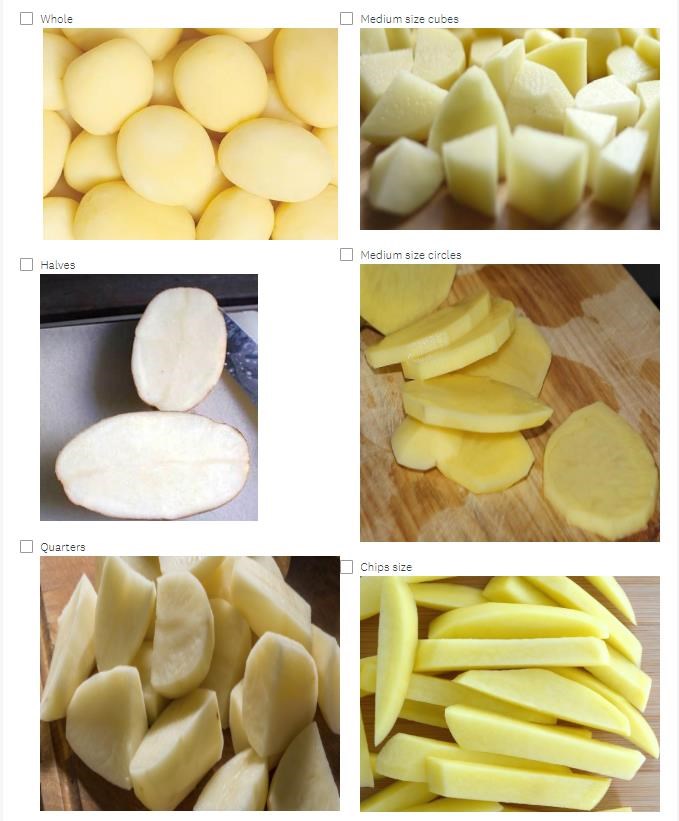 | | | |
|  | | | | | | | | | | |
|  | Wedges | | | | | |  | | Crisps size | |
| 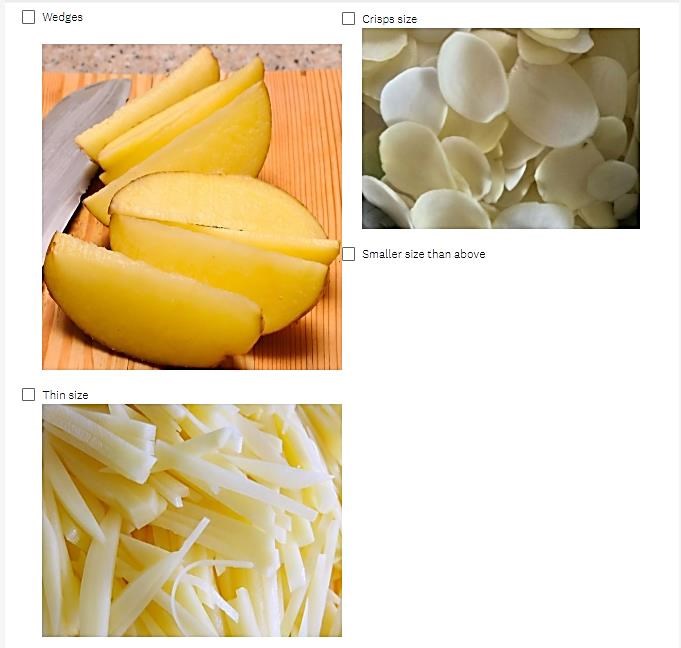 | | | | | | | 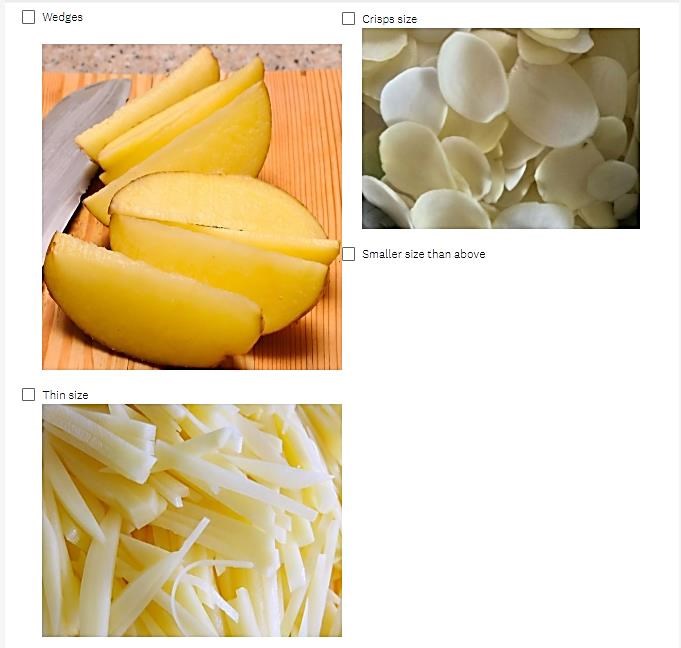 | | | |
|  | | | | | | | | | | |
|  | Thin size | | | | | |  | | Smaller size than above | |
| 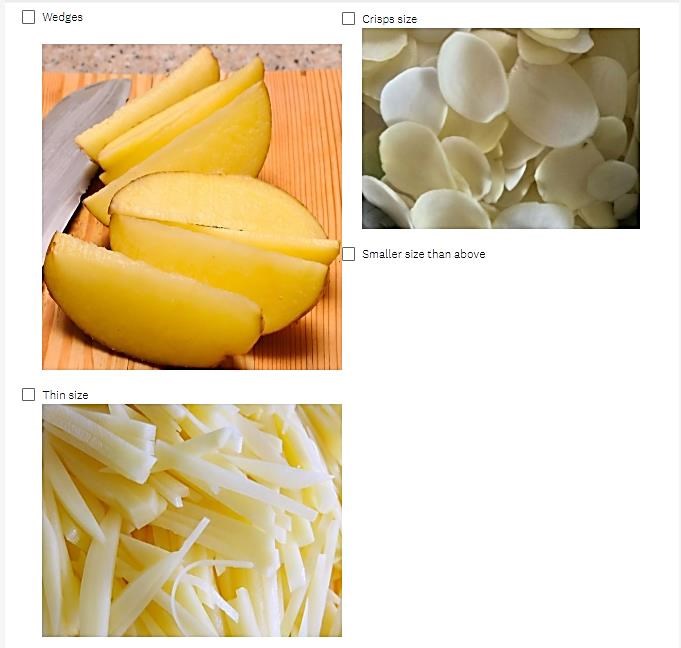 | | | | | | |  | | | |
|  |  |  |  |  |  |  |  | | Other (Please specify) | |
|  |  |  |  |  |  |  |  | | | |
|  | | | | | | | | | | |
| 1. **Do you soak peeled or cut potatoes before frying or roasting?** | | | | | | | | | | |
|  | | | | | | | | | | |
|  | Always | | | | | | | | | |
|  | | | | | | | | | | |
|  | Never (skip to question № 16) | | | | | | | | | |
|  | | | | | | | | | | |
|  | Usually | | | | | | | | | |
|  | | | | | | | | | | |
|  | Rarely | | | | | | | | | |
|  | | | | | | | | | | |
|  | Not sure (skip to question № 16) | | | | | | | | | |
|  | | | | | | | | | | |
| 1. **For what reason do you soak, or not soak raw potatoes?** | | | | | | | | | | |
| \|  \| \| --- \| | | | | | | | | | | |
|  | | | | | | | | | | |
| 1. **If you soak raw potatoes, what is the average time for soaking?** | | | | | | | | | | |
|  | | | | | | | | | | |
|  | 5 min or less | | | | | | | | | |
|  | | | | | | | | | | |
|  | 15 min | | | | | | | | | |
|  | | | | | | | | | | |
|  | 30 mins | | | | | | | | | |
|  | | | | | | | | | | |
|  | 1 hr. or more | | | | | | | | | |
|  | | | | | | | | | | |
|  | I don’t know the exact time | | | | | | | | | |
|  | | | | | | | | | | |
|  | Whatever is convenient between preparation and cooking | | | | | | | | | |
|  | | | | | | | | | | |
| 1. **Do you parboil the potatoes before cooking?** | | | | | | | | | | |
|  | | | | | | | | | | |
|  | Always | | | | | | | | | |
|  | | | | | | | | | | |
|  | Never (skip to question № 18) | | | | | | | | | |
|  | | | | | | | | | | |
|  | Usually | | | | | | | | | |
|  | | | | | | | | | | |
|  | Rarely | | | | | | | | | |
|  | | | | | | | | | | |
|  | Not sure (skip to question № 18) | | | | | | | | | |
|  | | | | | | | | | | |
| 1. **Why do you parboil the potatoes before cooking?** | | | | | | | | | | |
| \|  \| \| --- \| | | | | | | | | | | |
|  | | | | | | | | | | |
| 1. **When you cook pre-cooked foods (e.g. frozen chips), how do you estimate the cooking time?** | | | | | | | | | | |
|  | | | | | | | | | | |
|  | Visual assessment (i.e. of colour and texture) | | | | | | | | | |
|  | | | | | | | | | | |
|  | Abiding strictly to the instructed time on the label | | | | | | | | | |
|  | | | | | | | | | | |
|  | Tasting assessment | | | | | | | | | |
|  | | | | | | | | | | |
|  | Other (Please specify) | | | | | | | | | |
| \|  \| \| --- \| | | | | | | | | | | |
|  | | | | | | | | | | |
| 1. **What heat setting on your oven do you often select for roasting fresh potatoes?** | | | | | | | | | | |
|  | | | | | | | | | | |
| Gas oven fan/Electric oven | | | 1  1  (275°F/140°C) | | | 2  2  (300°F/150°C) | | 3  3  (325°F/170°C) | | 4  3  (350°F/180°C) |
|  |  |  | \|  \| \| --- \| | | | \|  \| \| --- \| | | \|  \| \| --- \| | | \|  \| \| --- \| |
|  |  |  | 5  (375°F/190°C) | | | 6  (400°F/200°C) | | 7  (425°F/220°C) | | 8  (450°F/230°C) |
|  |  |  | \|  \| \| --- \| | | | \|  \| \| --- \| | | \|  \| \| --- \| | | \|  \| \| --- \| |
|  |  |  | 9  (475°F/240°C) | | | I can’t remember | |  | | |
|  |  |  | \|  \| \| --- \| | | | \|  \| \| --- \| | |  |  |  |
|  | | | | | | | | | | |
| 1. **Which photo represents your preference for roasted potatoes?** | | | | | | | | | | |
|  | | | | | | | | | | |
|  | A | | | | 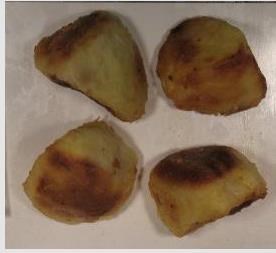 | | | | | |
|  |  |  |  |  |  |  |  |  |  |  |
| 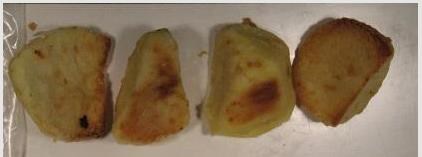 | | | | | | | | | | |
|  | B | | | |  | | | | | |
|  | | | | | | | | | | |
|  | | | | | | | | | | |
|  | | | | | | | | | | |
|  | C | | | | 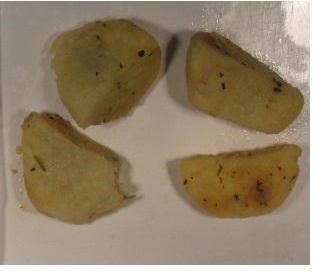 | | | | | |
|  |  |  |  |  |  |  |  |  |  |  |
|  | | | | | | | | | | |
|  | D | | | | I do not cook roasted potatoes at all. | | | | | |
|  | | | | | | | | | | |
| 1. **How do you normally cook chips?** | | | | | | | | | | |
|  | | | | | | | | | | |
|  | Deep-frying | | | | | | | | | |
|  | | | | | | | | | | |
|  | Air fryer | | | | | | | | | |
|  | | | | | | | | | | |
|  | Pan/shallow | | | | | | | | | |
|  | | | | | | | | | | |
|  | Roasting | | | | | | | | | |
|  | | | | | | | | | | |
|  | Grilled | | | | | | | | | |
|  | | | | | | | | | | |
|  | Microwaved | | | | | | | | | |
|  | | | | | | | | | | |
|  | I do not cook chips | | | | | | | | | |
|  | | | | | | | | | | |
|  | Other (Please specify) | | | | | | | | | |
| \|  \| \| --- \| | | | | | | | | | | |
|  | | | | | | | | | | |
| 1. **Which photo represents your preference for chips?** | | | | | | | | | | |
|  | | | | | | | | | | |
|  | A | | | | 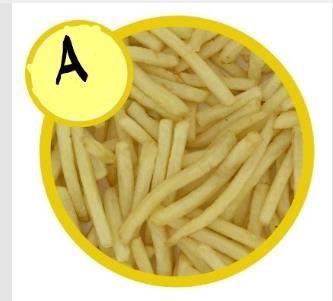 | | | | | |
|  | | | | | | | | | | |
|  | B | | | | 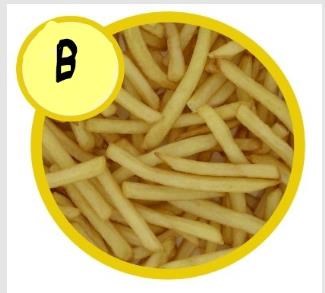 | | | | | |
|  |  |  |  |  |  |  |  |  |  |  |
|  | | | | | | | | | | |
|  | | | | | | | | | | |
|  | | | | | | | | | | |
|  | | | | | | | | | | |
|  | C | | | | 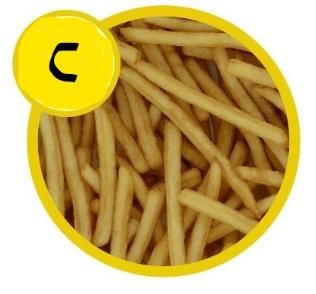 | | | | | |
|  |  |  |  |  |  |  |  |  |  |  |
|  | | | | | | | | | | |
|  | D | | | | I do not like / eat at all. | | | | | |
|  | | | | | | | | | | |
| 1. **For what reasons do you have these colour preferences?** | | | | | | | | | | |
|  | | | | | | | | | | |
|  | | | | | | | | | | |
|  | | | | | | | | | | |
| 1. **How often do you eat chips or roasted potatoes?** | | | | | | | | | | |
|  | | | | | | | | | | |
|  | Daily  2-3 times per week | | | | | | | | | |
|  |  |  |  |  |  |  |  |  |  |  |
|  | | | | | | | | | | |
|  | Once a week | | | | | | | | | |
|  | | | | | | | | | | |
|  | Once a month | | | | | | | | | |
|  | | | | | | | | | | |
|  | Never | | | | | | | | | |
|  | | | | | | | | | | |
| 1. **What type of bread do you usually buy? ‘tick all that apply’** | | | | | | | | | | |
|  | | | | | | | | | | |
|  | White bread | | | | | | | | | |
|  | | | | | | | | | | |
|  | Brown bread | | | | | | | | | |
|  | | | | | | | | | | |
|  | Whole wheat bread | | | | | | | | | |
|  | | | | | | | | | | |
|  | I do not use bread | | | | | | | | | |
|  | | | | | | | | | | |
|  | Other (Please specify) | | | | | | | | | |
| \|  \| \| --- \| | | | | | | | | | | |
|  | | | | | | | | | | |
| 1. **Which photo represents your preference for toasting bread?** | | | | | | | | | | |
|  | | | | | | | | | | |
|  | A | | | 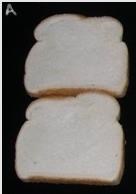 | | | | | | |
|  |  | | |  |  |  |  |  |  |  |
|  |  | | |  |  |  |  |  |  |  |
|  | | | | | | | | | | |
|  | B | | | 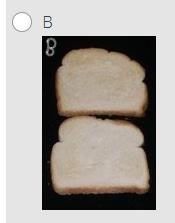 | | | | | | |
|  |  | | |  |  |  |  |  |  |  |
|  |  | | |  |  |  |  |  |  |  |
|  | | | | | | | | | | |
|  | C | | | 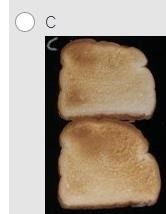 | | | | | | |
|  |  | | |  |  |  |  |  |  |  |
|  |  | | |  |  |  |  |  |  |  |
|  | | | | | | | | | | |
|  | D | | | 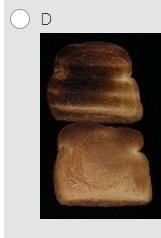 | | | | | | |
|  |  | | |  |  |  |  |  |  |  |
|  |  | | |  |  |  |  |  |  |  |
|  | | | | | | | | | | |
|  | Other (Please specify) | | | | | | | | | |
| \|  \| \| --- \| | | | | | | | | | | |
|  | | | | | | | | | | |
| 1. **Why do you like your preference for how well-toasted bread you like?** | | | | | | | | | | |
| \|  \| \| --- \| | | | | | | | | | | |
|  | | | | | | | | | | |
| 1. **How many slices of bread do you usually eat per day?** | | | | | | | | | | |
|  | | | | | | | | | | |
|  | Rarely | | | | | | | | | |
|  | | | | | | | | | | |
|  | 1-2 slices/day | | | | | | | | | |
|  | | | | | | | | | | |
|  | 3-5 slices/day | | | | | | | | | |
|  | | | | | | | | | | |
|  | More than 5 slices/day | | | | | | | | | |
|  | | | | | | | | | | |
|  | Other (Please specify) | | | | | | | | | |
| \|  \| \| --- \| | | | | | | | | | | |
|  | | | | | | | | | | |
| 1. **Which type of coffee do you usually drink?** | | | | | | | | | | |
|  | | | | | | | | | | |
|  | Instant coffee | | | | | | | | | |
|  | | | | | | | | | | |
|  | Roasted/ground coffee | | | | | | | | | |
|  | | | | | | | | | | |
|  | Coffee substitutes | | | | | | | | | |
|  | | | | | | | | | | |
|  | I don’t drink coffee | | | | | | | | | |
|  | | | | | | | | | | |
|  | Other (Please specify) | | | | | | | | | |
| \|  \| \| --- \| | | | | | | | | | | |
|  | | | | | | | | | | |
| 1. **How many cups of coffee do you drink daily?** | | | | | | | | | | |
|  | | | | | | | | | | |
|  | | | | | | | | | | |
|  | 1 cup | | | | | | | | | |
|  | | | | | | | | | | |
|  | 2 cups | | | | | | | | | |
|  | | | | | | | | | | |
|  | 3 cups | | | | | | | | | |
|  | | | | | | | | | | |
|  | 4 cups | | | | | | | | | |
|  | | | | | | | | | | |
|  | 5 cups | | | | | | | | | |
|  | | | | | | | | | | |
|  | More than 5 cups  I don’t drink coffee | | | | | | | | | |
|  |  |  |  |  |  |  |  |  |  |  |
|  | | | | | | | | | | |
| 1. **Have you previously heard about the formation of a harmful compound when some carbohydrate foods (e.g. bread, potatoes) are overheated to brown colour appearance?** | | | | | | | | | | |
|  | | | | | | | | | | |
|  | Yes | | | | | | | | | |
|  | | | | | | | | | | |
|  | No (skip to question № 34) | | | | | | | | | |
|  | | | | | | | | | | |
| 1. **What do you think are the negative health effects from this a harmful compound?** | | | | | | | | | | |
|  | | | | | | | | | | |
|  | I do not know | | | | | | | | | |
|  | | | | | | | | | | |
|  | I know that (Please specified in the below box) | | | | | | | | | |
|  | | | | | | | | | | |
|  | The negative health effects may be: | | | | | | | | | |
| \|  \| \| --- \| | | | | | | | | | | |
|  | | | | | | | | | | |
| 1. **From what source did you get your information about this harmful compound? (Tick as many as are relevant)** | | | | | | | | | | |
|  | | | | | | | | | | |
|  | TV | | | | | | | | | |
|  | | | | | | | | | | |
|  | Internet/ social media | | | | | | | | | |
|  | | | | | | | | | | |
|  | Journal/Magazine | | | | | | | | | |
|  | | | | | | | | | | |
|  | From person (Family/friends) | | | | | | | | | |
|  | | | | | | | | | | |
|  | School/College | | | | | | | | | |
|  | | | | | | | | | | |
|  | Food Safety Authority of Lebanon | | | | | | | | | |
|  | | | | | | | | | | |
|  | Other (Please specify) | | | | | | | | | |
|  | | | | | | | | | | |
| 1. **“ACRYLAMIDE is a harmful compound produced when some carbohydrate foods become brown due to overheating” , should food packaging contain information about the content and safe level of ACRYLAMIDE ( for example the maximum daily limit)?** | | | | | | | | | | |
|  | | | | | | | | | | |
|  | Yes, definitely | | | | | | | | | |
|  | | | | | | | | | | |
|  | Definitely, not | | | | | | | | | |
|  | | | | | | | | | | |
|  | I don’t have a strong opinion either. | | | | | | | | | |
|  | | | | | | | | | | |
| 1. **In your opinion, should food packaging contain information about the potential formation of ACRYLAMIDE in the product if the cooking instructions are not properly followed?** | | | | | | | | | | |
|  | | | | | | | | | | |
|  | | Yes, definitely | | | | | | | | |
|  | | | | | | | | | | |
|  | | Definitely, not | | | | | | | | |
|  | | | | | | | | | | |
|  | | I don’t have a strong opinion either. | | | | | | | | |
|  | | | | | | | | | | |
| 1. **In your opinion, if a product’s label listed ACRYLAMIDE at a safe level (or below the recommended daily limit), would you buy the product?** | | | | | | | | | | |
|  | | | | | | | | | | |
|  | | Yes, definitely | | | | | | | | |
|  | | | | | | | | | | |
|  | | Definitely, not | | | | | | | | |
|  | | | | | | | | | | |
|  | | Not sure | | | | | | | | |
|  | | | | | | | | | | |
|  | | | | | | | | | | |
