# Supplementary material for: An exploratory study to assess the knowledge, attitudes and practices of Lebanese residents towards acrylamide
Source: PLoS One. 2024 Apr 16;19(4):e0300617. doi: 10.1371/journal.pone.0300617 (PMC11020536; doi:10.1371/journal.pone.0300617)
Supplement: S2 Appendix — (DOCX) [file pone.0300617.s002.docx]

المعرفة والمواقف والممارسات المتعلقة بمادة ألكريلميد في الطعام بين سكان لبنان

| 1. **مكان الإقامة** | | | | | | |
| --- | --- | --- | --- | --- | --- | --- |
|  | | | | | | |
|  | أقيم في لبنان | | | | | |
|  | | | | | | |
|  | غير مقيم في لبنان | | | | | |
|  | | | | | | |
| 1. **هل يتجاوز عمرك 18 سنة؟** | | | | | | |
|  | | | | | | |
|  | نعم | | | | | |
|  | | | | | | |
|  | كلا | | | | | |
|  | | | | | | |
| \| 1. **ما هو جنسك؟** \| \| \| \| \| \| \| \| \| \| \| --- \| --- \| --- \| --- \| --- \| --- \| --- \| --- \| --- \| --- \| \|  \| \| \| \| \| \| \| \| \| \| \|  \| \| \| \| \| ذكر \| \| \| \| \| \|  \| \| \| \| \| \| \| \| \| \| \|  \| \| \| \| \| أنثى \| \| \| \| \| \|  \| \| \| \| \| \| \| \| \| \| \| 1. **الوضع العائلي** \| \| \| \| \| \| \| \| \| \| \|  \| \| \| \| \| \| \| \| \| \| \|  \| \| \| \| \| عازب \| \| \| \| \| \|  \| \| \| \| \| \| \| \| \| \| \|  \| \| \| \| \| \| \| \| \| \| \|  \| \| \| \| \| متأهل دون أولاد \| \| \| \| \| \|  \| \| \| \| \| \| \| \| \| \| \|  \| \| \| \| \| متأهل ولك أولاد \| \| \| \| \| \|  \| \| \| \| \| \| \| \| \| \| \|  \| \| \| \| \| غير ذلك \| \| \| \| \| \| 1. **ما هو المستوى التعليمي الذي أكملته؟** \| \| \| \| \| \| \| \| \| \| \|  \| \| \| \| \| \| \| \| \| \| \|  \| \| \| \| \| شهادة الثانوية العامة \| \| \| \| \| \|  \| \| \| \| \| \| \| \| \| \| \|  \| \| \| \| \| شهادة البكالوريوس \| \| \| \| \| \|  \| \| \| \| \| \| \| \| \| \| \|  \| \| \| \| \| شهادة الماجستير/ الدكتوراه \| \| \| \| \| \|  \| \| \| \| \| \| \| \| \| \| \|  \| \| \| \| \| لا مؤهلات \| \| \| \| \| \| 1. **متوسط دخل الأسرة هو** \| \| \| \| \| \| \| \| \| \| \|  \| \| \| \| \| \| \| \| \| \| \|  \| \| \| \| \| أقل من 100$ \| \| \| \| \| \|  \| \| \| \| \| \| \| \| \| \| \|  \| \| \| \| \| ما بين 100 و 500 $ \| \| \| \| \| \|  \| \| \| \| \| \| \| \| \| \| \|  \| \| \| \| \| أكثر من 500 $ \| \| \| \| \| \| 1. **ما هي فئتك العمرية؟** \| \| \| \| \| \| \| \| \| \| \|  \| \| \| \| \| \| \| \| \| \| \|  \| \| \| \| \| تحت 18 \| \| \| \| \| \|  \| \| \| \| \| \| \| \| \| \| \|  \| \| \| \| \| 18-24 \| \| \| \| \| \|  \| \| \| \| \| \| \| \| \| \| \|  \| \| \| \| \| 25-34 \| \| \| \| \| \|  \| \| \| \| \| \| \| \| \| \| \|  \| \| \| \| \| 35-44 \| \| \| \| \| \|  \| \| \| \| \| \| \| \| \| \| \|  \| \| \| \| \| 45-54 \| \| \| \| \| \|  \| \| \| \| \| \| \| \| \| \| \|  \| \| \| \| \| 55-64 \| \| \| \| \| \|  \| \| \| \| \| \| \| \| \| \| \|  \| \| \| \| \| فوق 65 \| \| \| \| \| \| 1. **ما هي المنطقة التي تقيم فيها؟** \| \| \| \| \| \| \| \| \| \| \|  \| \| \| \| \| \| \| \| \| \| \|  \| \| \| \| \| المدينة / البلدة \| \| \| \| \| \|  \| \| \| \| \| \| \| \| \| \| \|  \| \| \| \| \| الريف \| \| \| \| \|  1. **هل سمعت من قبل عن مادة الأكريلاميد في الطعام؟** | | | | | | |
|  | | | | | | |
|  | نعم | | | | | |
|  | | | | | | |
|  | كلا (انتقل إلى السؤال رقم 7) | | | | | |
|  | | | | | | |
| 1. **أي من الأطعمة التالية يمكن أن تساهم في التعرض العالي لمادة الأكريلاميد؟ (ضع إشارة في المربعات المناسبة).** | | | | | | |
|  | | | | | | |
|  | خضروات ورقية | | | | | |
|  | | | | | | |
|  | الفواكه | | | | | |
|  | | | | | | |
|  | الحليب | | | | | |
|  | | | | | | |
|  | اللحوم | | | | | |
|  | | | | | | |
|  | الأسماك | | | | | |
|  | | | | | | |
|  | الخبز | | | | | |
|  | | | | | | |
|  | البيض | | | | | |
|  | | | | | | |
|  | البطاطا | | | | | |
|  | | | | | | |
|  | الدواجن | | | | | |
|  | | | | | | |
|  | القهوة | | | | | |
|  | | | | | | |
|  | البسكويت / الرقائق / الحبوب | | | | | |
|  | | | | | | |
|  | | | | | | |
|  | الحبوب | | | | | |
|  | | | | | | |
| 1. **لماذا تعتقد ذلك؟** | | | | | | |
|  | | | | | | |
|  | لا أعلم | | | | | |
|  | | | | | | |
|  | إنني أختار هذه الأطعمة لأنه: (نرجو التحديد في المربع أدناه) | | | | | |
|  | | | | | | |
| أعتقد: | | | | | | |
| \|  \| \| --- \| | | | | | | |
|  | | | | | | |
| 1. **وفق أي ظروف تعتقد أن مادة الأكريلاميد يمكن أن تتشكل في الأطعمة؟ (ضع إشارة في المربعات المناسبة).** | | | | | | |
|  | | | | | | |
|  | أثناء التجميد | | | | | |
|  | | | | | | |
|  | أثناء التحضير (على سبيل المثال التقشير، التقطيع، إلخ ...) | | | | | |
|  | | | | | | |
|  | في البراد | | | | | |
|  | | | | | | |
|  | أثناء التخزين بدرجة حرارة الغرفة | | | | | |
|  | | | | | | |
|  | أثناء الطبخ بدرجة حرارة عالية (مثل القلي أو التحميص أو الخبز) | | | | | |
|  | | | | | | |
|  | بعد الأكل/ الهضم | | | | | |
|  | | | | | | |
|  | لا أعلم | | | | | |
|  | | | | | | |
| 1. **أين تخزن البطاطا النيئة عادةً؟** | | | | | | |
|  | | | | | | |
|  | في البراد | | | | | |
|  | | | | | | |
|  | في الثلاجة | | | | | |
|  | | | | | | |
|  | في خزانة أو غرفة المؤن بدرجة حرارة الغرفة | | | | | |
|  | | | | | | |
|  | لا أستخدم البطاطا النيئة على الإطلاق (انتقل إلى السؤال رقم 18) | | | | | |
|  | | | | | | |
|  | غير ذلك (نرجو التحديد) | | | | | |
| \|  \| \| --- \| | | | | | | |
|  | | | | | | |
| 1. **لأي سبب تقوم بتخزين البطاطا النيئة هناك؟** | | | | | | |
| \|  \| \| --- \| | | | | | | |
|  | | | | | | |
| 1. **بعد كم من الوقت من تاريخ الشراء يمكنك تخزين البطاطا النيئة عادةً؟** | | | | | | |
|  | | | | | | |
|  | 0-3 أيام | | | | | |
|  | | | | | | |
|  | 4-6 أيام | | | | | |
|  | | | | | | |
|  | 7-9 أيام | | | | | |
|  | | | | | | |
|  | لمدة أسبوعين | | | | | |
|  | | | | | | |
|  | أكثر من أسبوعين | | | | | |
|  | | | | | | |
|  | لا أستطيع أن أتذكر | | | | | |
|  | | | | | | |
| 1. **هل تقشر البطاطا عادةً قبل الطهي؟** | | | | | | |
|  | | | | | | |
|  | دائماً | | | | | |
|  | | | | | | |
|  | أبداً (انتقل إلى السؤال رقم 12) | | | | | |
|  | | | | | | |
|  | عادةً | | | | | |
|  | | | | | | |
|  | نادراً | | | | | |
|  | | | | | | |
|  | لست متأكداً | | | | | |
|  | | | | | | |
| 1. **هل تغسل البطاطا عادةً بعد تقشيرها؟** | | | | | | |
|  | | | | | | |
|  | دائماً | | | | | |
|  | | | | | | |
|  | أبداً | | | | | |
|  | | | | | | |
|  | عادةً | | | | | |
|  | | | | | | |
|  | نادراً | | | | | |
|  | | | | | | |
|  | لست متأكداً | | | | | |
|  | | | | | | |
|  | | | | | | |
|  | | | | | | |
|  | | | | | | |
|  | | | | | | |
|  | | | | | | |
|  | | | | | | |
|  | | | | | | |
|  | | | | | | |
|  | | | | | | |
|  | | | | | | |
|  | | | | | | |
| 1. **ما هو الحجم الذي تقطع به البطاطا التي تنوي تحميصها؟ (ضع إشارة في المربعات المناسبة)؟** | | | | | | |
|  | | | | | | |
|  | حبة كاملة | | | |  | 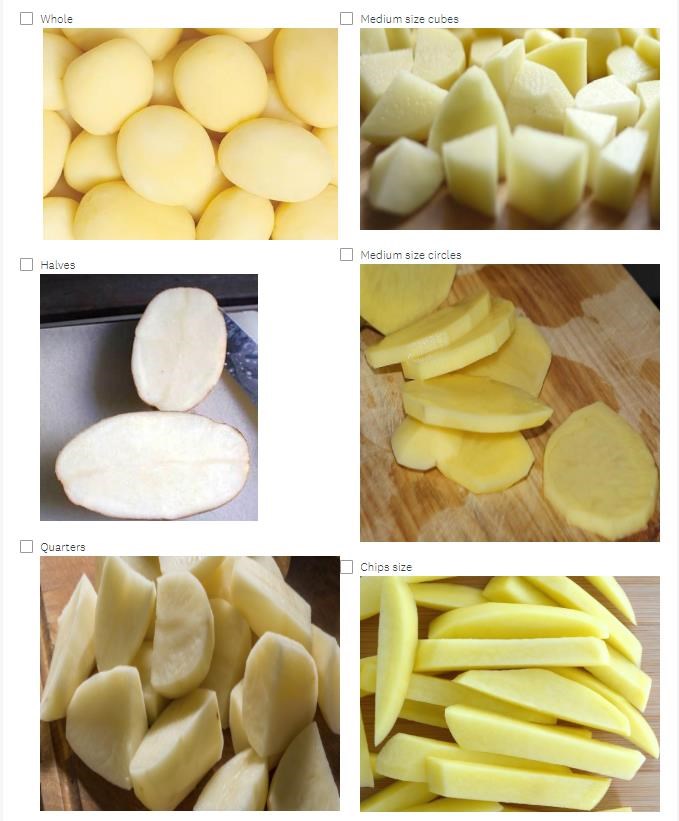مكعبات حجم وسط |
| 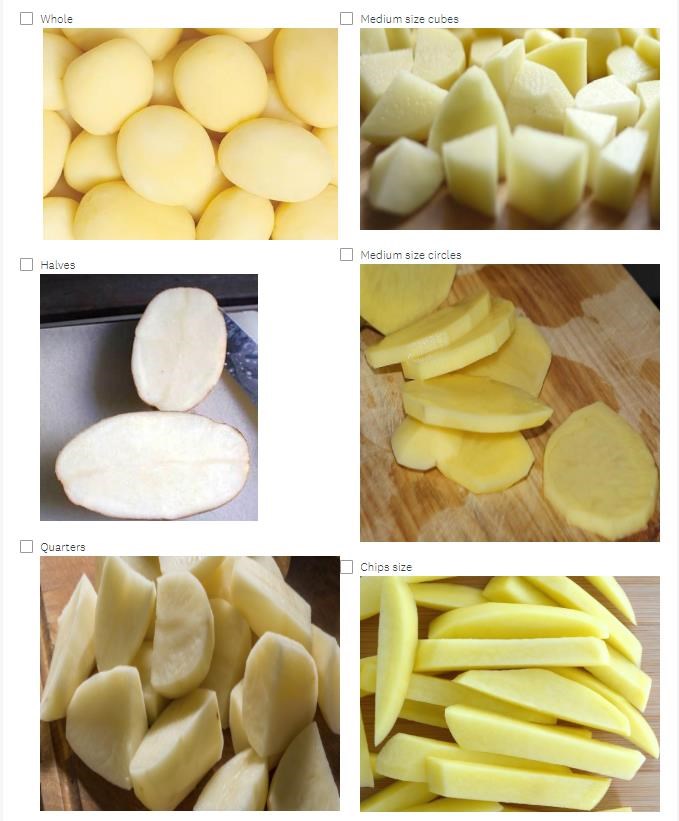 | | | | |  | |
|  | | | | | | |
|  | أنصاف | | | |  | 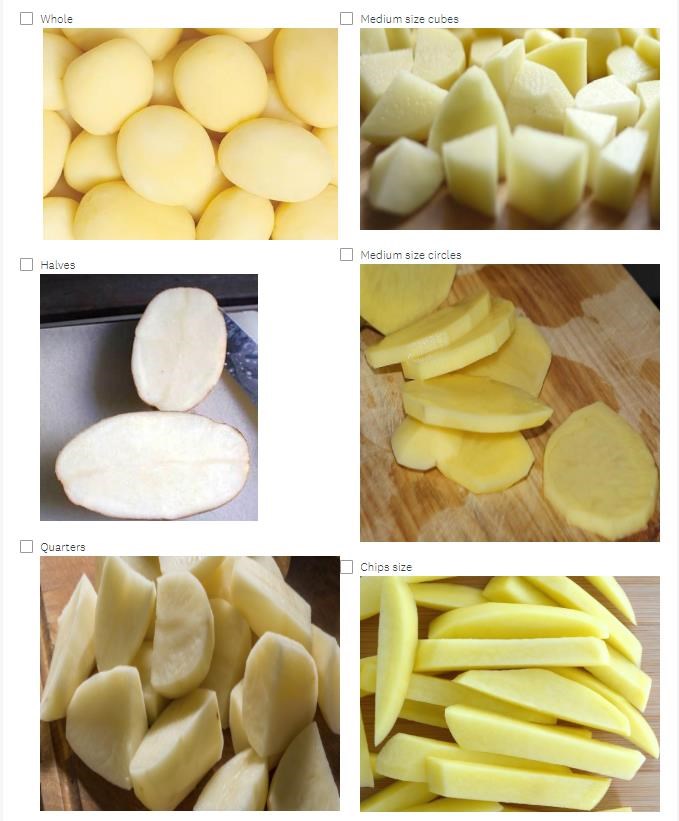دوائر حجم وسط |
| 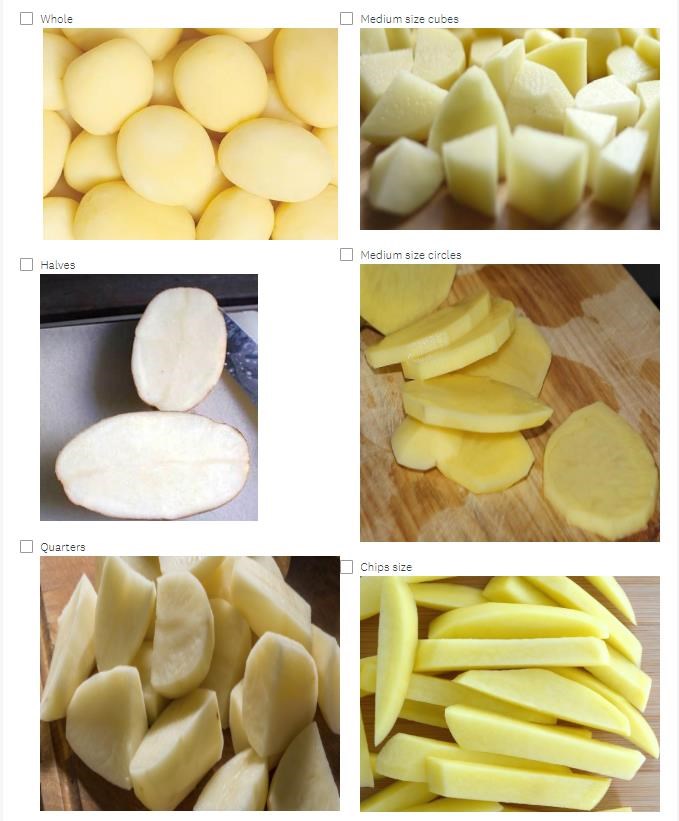 | | | | | 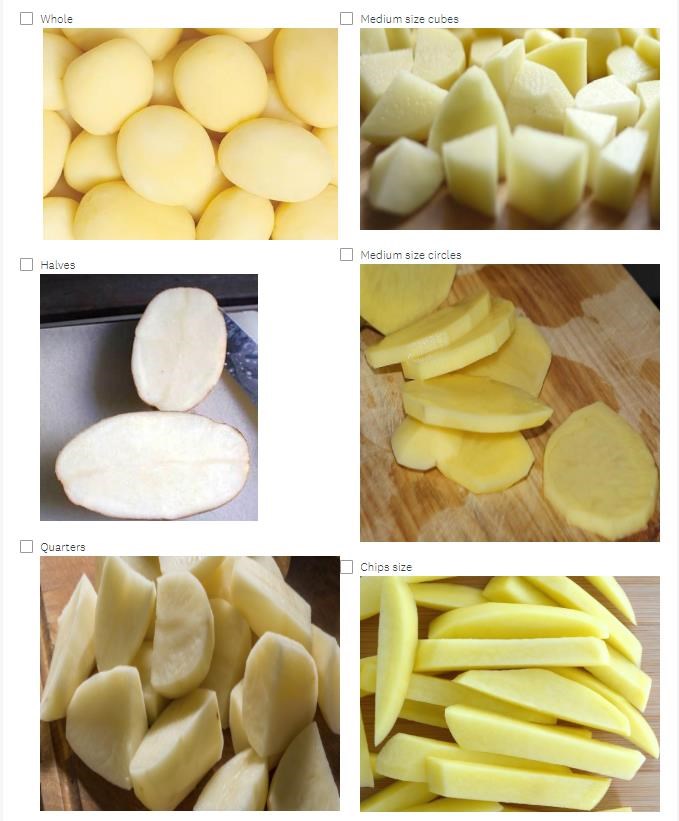 | |
|  | | | | | | |
|  | أرباع | | | |  | حجم الرقائق |
| 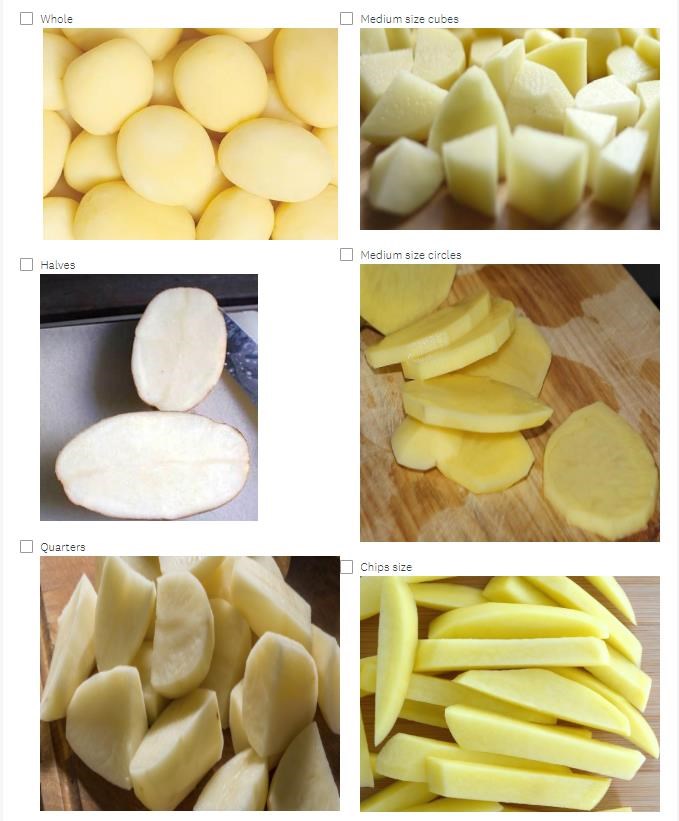 | | | | | 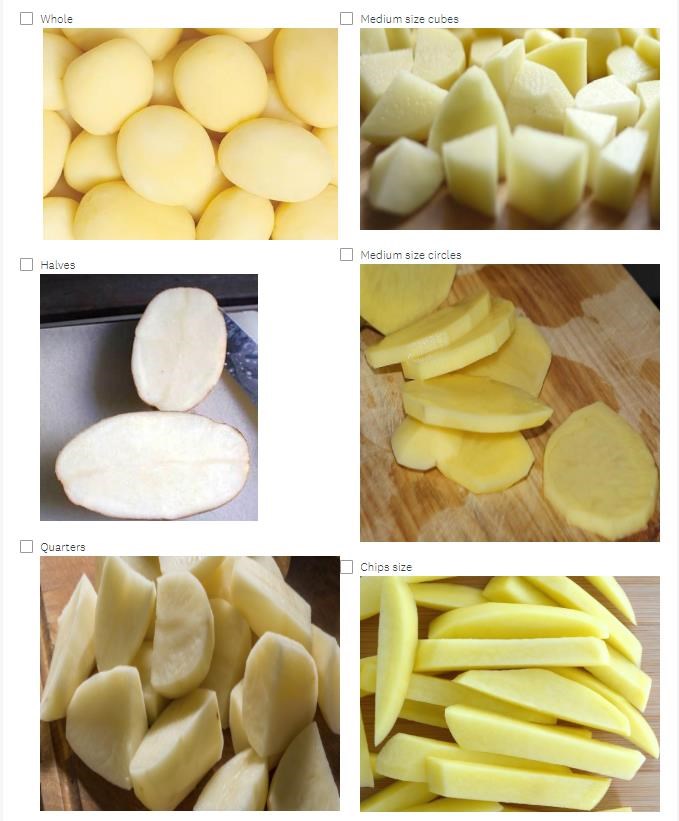 | |
|  | | | | | | |
|  | ويدجز | | | |  | 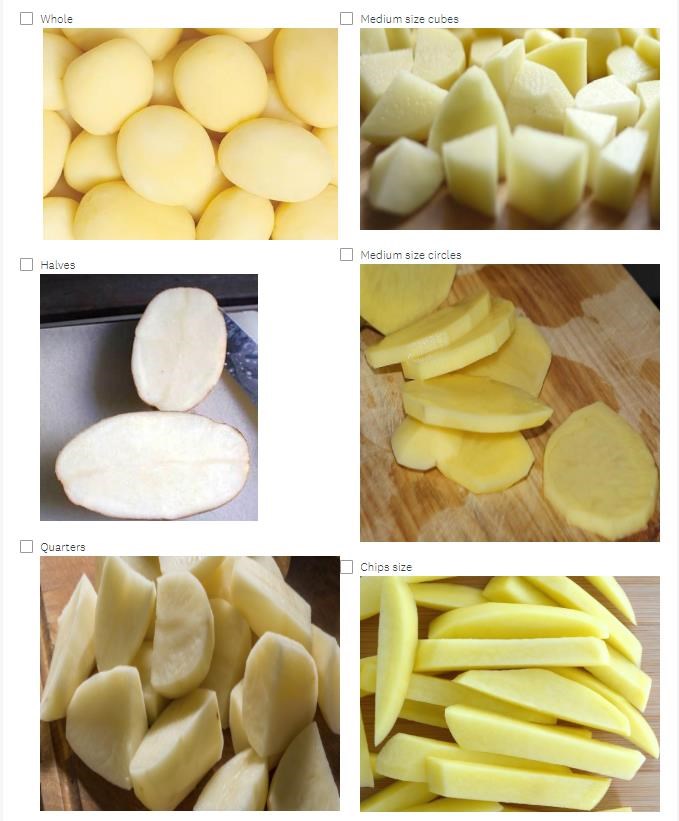حجم رقائق البطاطا |
| 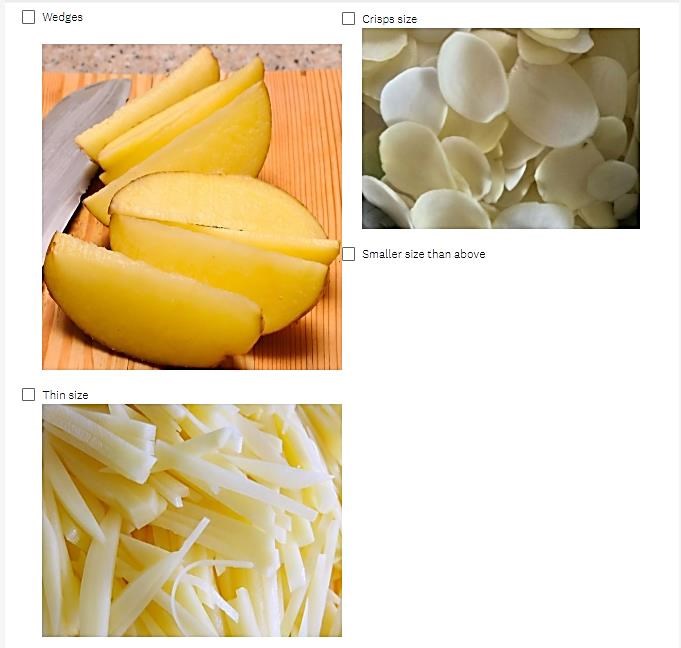 | | | | | 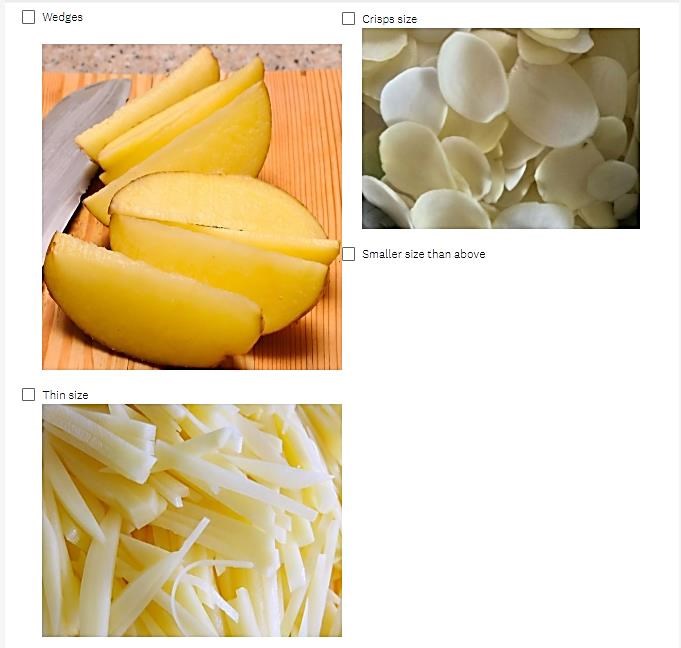 | |
|  | | | | | | |
|  | حجم رفيع | | | |  | حجم أصغر مما ورد أعلاه |
| 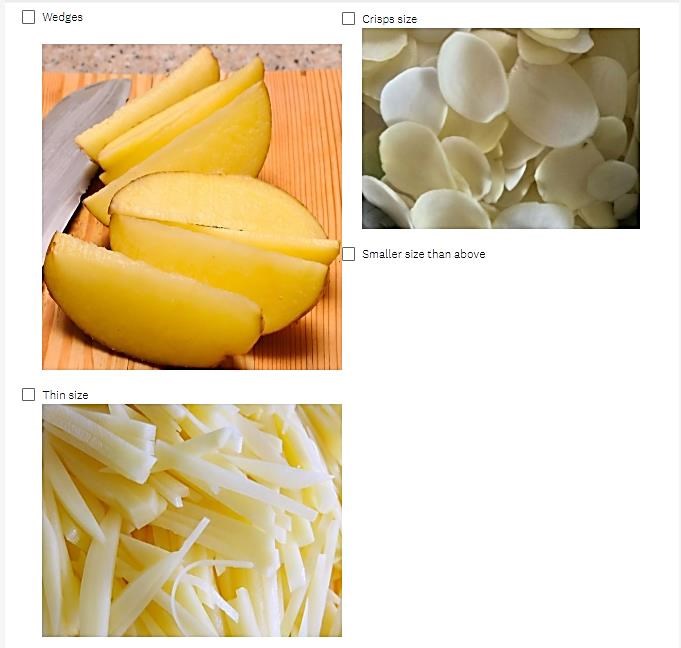 | | | | |  | |
|  |  |  |  |  |  | غير ذلك (نرجو التحديد) |
|  |  |  |  |  |  | |
|  | | | | | | |
| 1. **هل تنقع البطاطا المقشرة أو المقطعة قبل قليها أو تحميرها؟** | | | | | | |
|  | | | | | | |
|  | دائماً | | | | | |
|  | | | | | | |
|  | أبداً (انتقل إلى السؤال رقم 16) | | | | | |
|  | | | | | | |
|  | عادةً | | | | | |
|  | | | | | | |
|  | نادراً | | | | | |
|  | | | | | | |
|  | لست متأكداً (انتقل إلى السؤال رقم 16) | | | | | |
|  | | | | | | |
| 1. **ما سبب نقع البطاطا النيئة أو عدم نقعها؟** | | | | | | |
| \|  \| \| --- \| | | | | | | |
|  | | | | | | |
| 1. **إذا قمت بنقع البطاطا النيئة، ما هو متوسط وقت نقعها؟** | | | | | | |
|  | | | | | | |
|  | 5 دقائق أو أقل | | | | | |
|  | | | | | | |
|  | 15 دقيقة | | | | | |
|  | | | | | | |
|  | 30 دقيقة | | | | | |
|  | | | | | | |
|  | ساعة واحدة أو أكثر | | | | | |
|  | | | | | | |
|  | لا أعرف الوقت بالتحديد | | | | | |
|  | | | | | | |
|  | كل ما هو مناسب بين وقت التحضير والطهي | | | | | |
|  | | | | | | |
| 1. **هل تقوم بسلق البطاطا قبل طهيها؟** | | | | | | |
|  | | | | | | |
|  | دائماً | | | | | |
|  | | | | | | |
|  | أبداً (انتقل إلى السؤال رقم 18) | | | | | |
|  | | | | | | |
|  | كالمعتاد | | | | | |
|  | | | | | | |
|  | نادراً | | | | | |
|  | | | | | | |
|  | لست متأكداً (انتقل إلى السؤال رقم 18) | | | | | |
|  | | | | | | |
| 1. **لماذا تقوم بسلق البطاطا قبل طهيها؟** | | | | | | |
| \|  \| \| --- \| | | | | | | |
|  | | | | | | |
| 1. **عند طهي الأطعمة المطبوخة مسبقا (مثل البطاطا المقلية المجمدة)، كيف تقدر وقت طهيها؟** | | | | | | |
|  | | | | | | |
|  | أقيمه بصرياً (على سبيل المثال: اللون واالملمس) | | | | | |
|  | | | | | | |
|  | ألتزام بصرامة بالوقت المحدد على الملصق | | | | | |
|  | | | | | | |
|  | أقيمه بتذوقه | | | | | |
|  | | | | | | |
|  | غير ذلك (نرجو التحديد) | | | | | |
| \|  \| \| --- \| | | | | | | |
|  | | | | | | |
| 1. **ما هي درجة حرارة الفرن التي تختارها غالباً لتحمير البطاطا الطازجة؟**  \|  \| 1  (120)  (275°ف/140°س) \| 2  (130)  (300°ف/150°س) \| 3  (150)  (325°ف/180°س) \| 4  (160)  (350°ف/180°س) \| 5  (170)  (375°ف/190°س) \| 6  (180)  (400°ف/200°س) \| 7  (200)  (425°ف/220°س) \| 8  (210)  (450°ف/230°س) \| 9  (220)  (475°ف/240°س) \| لا أذكر \| \| --- \| --- \| --- \| --- \| --- \| --- \| --- \| --- \| --- \| --- \| --- \| \| فرن غاز  فرن مروحة/كهربائي \|  \|  \|  \|  \|  \|  \|  \|  \|  \|  \| | | | | | | |
|  | | | | | | |
| 1. **ما هي الصورة التي تمثل البطاطا المحمرة المفضلة لديك؟** | | | | | | |
|  | | | | | | |
|  | أ | | | 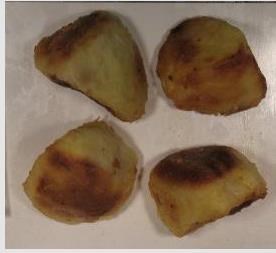 | | |
|  |  |  |  |  |  |  |
|  | | | | | | |
|  | ب | | | 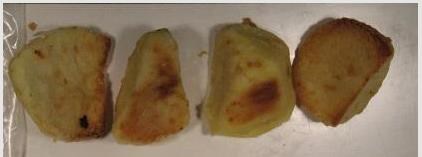 | | |
|  |  |  |  |  |  |  |
|  | | | | | | |
|  | ج | | | 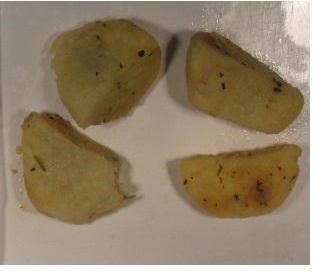 | | |
|  |  |  |  |  |  |  |
|  | | | | | | |
|  | د | | | لا أقوم بإعداد البطاطا المحمرة على الإطلاق. | | |
|  | | | | | | |
| 1. **كيف تطهو رقائق البطاطا عادةً؟** | | | | | | |
|  | | | | | | |
|  | مقلي قلياً عميقاً | | | | | |
|  | | | | | | |
|  | بالمقلاة الهوائية (Air Fryer) | | | | | |
|  | | | | | | |
|  | بالمقلاة قليلة الزيت | | | | | |
|  | | | | | | |
|  | بتحميصها | | | | | |
|  | | | | | | |
|  | بشويها | | | | | |
|  | | | | | | |
|  | بالمايكرويف | | | | | |
|  | | | | | | |
|  | لا أحضر رقائق البطاطا على الإطلاق | | | | | |
|  | | | | | | |
|  | غير ذلك (نرجو التحديد) | | | | | |
| \|  \| \| --- \| | | | | | | |
|  | | | | | | |
| 1. **ما الصورة التي تمثل رقائق البطاطا المفضلة لديك؟** | | | | | | |
|  | | | | | | |
|  | أ | | | 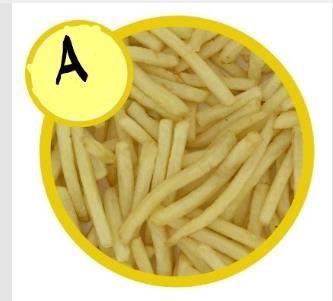 | | |
|  |  |  |  |  |  |  |
|  | | | | | | |
|  | ب | | | 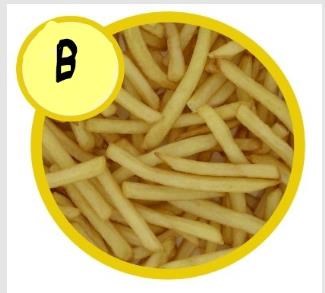 | | |
|  |  |  |  |  |  |  |
|  | | | | | | |
|  | | | | | | |
|  | | | | | | |
|  | | | | | | |
|  | | | | | | |
|  | | | | | | |
|  | | | | | | |
|  | ج | | | 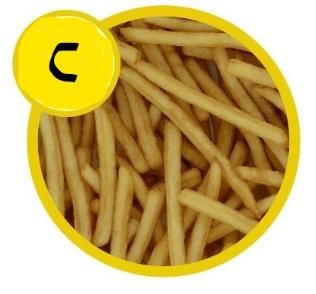 | | |
|  |  |  |  |  |  |  |
|  | | | | | | |
|  | د | | | لا أحب تناولها على الإطلاق. | | |
|  | | | | | | |
| 1. **لأي أسباب تفضل لون البطاطا هذا؟** | | | | | | |
|  | | | | | | |
|  | للبطاطا المحمرة | | | \|  \| \| --- \| | | |
|  | | | | | | |
|  | للرقائق | | | \|  \| \| --- \| | | |
|  | | | | | | |
| 1. **كم مرة تتناول رقائق البطاطا أو البطاطا المحمرة؟** | | | | | | |
|  | | | | | | |
|  | يومياً | | | | | |
|  | 2-3 مرات أسبوعياً | | | | | |
|  | | | | | | |
|  | مرة في الأسبوع | | | | | |
|  | | | | | | |
|  | مرة في الشهر | | | | | |
|  | | | | | | |
|  | لا أتناولها مطلقاً | | | | | |
|  | | | | | | |
| 1. **ما نوع الخبز الذي تشتريه عادة؟ 'ضع إشارة في المربع المناسب لذلك'** | | | | | | |
|  | | | | | | |
|  | الخبز الأبيض | | | | | |
|  | | | | | | |
|  | الخبز الأسمر | | | | | |
|  | | | | | | |
|  | خبز الدقيق الكامل | | | | | |
|  | | | | | | |
|  | | | | | | |
|  | لا أتناول الخبز | | | | | |
|  | | | | | | |
|  | غير ذلك (نرجو التحديد) | | | | | |
| \|  \| \| --- \| | | | | | | |
|  | | | | | | |
| 1. **ما الصورة التي تمثل الخبز المحمص المفضل لديك؟** | | | | | | |
|  | | | | | | |
|  | أ | | 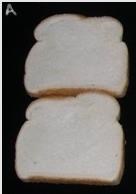 | | | |
|  |  | |  |  |  |  |
|  |  | |  |  |  |  |
|  | | | | | | |
|  | ب | | 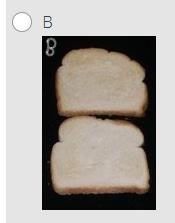 | | | |
|  |  | |  |  |  |  |
|  |  | |  |  |  |  |
|  | | | | | | |
|  | ج | | 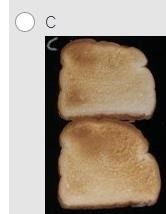 | | | |
|  |  | |  |  |  |  |
|  |  | |  |  |  |  |
|  | | | | | | |
|  | د | | 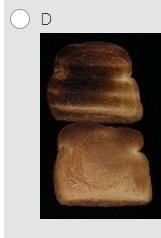 | | | |
|  |  | |  |  |  |  |
|  |  | |  |  |  |  |
|  | | | | | | |
|  | غير ذلك (نرجو التحديد) | | | | | |
| \|  \| \| --- \| | | | | | | |
|  | | | | | | |
| 1. **لماذا يعجبك تفضيلك لمدى تحميص الخبز الذي تحبه؟** | | | | | | |
| \|  \| \| --- \| | | | | | | |
|  | | | | | | |
| 1. **كم عدد أرغفة الخبز التي تتناولها في اليوم الواحد عادةً؟** | | | | | | |
|  | | | | | | |
|  | نادراً | | | | | |
|  | | | | | | |
|  | 1-2 / اليوم | | | | | |
|  | | | | | | |
|  | 3- 5 / اليوم | | | | | |
|  | | | | | | |
|  | أكثر من 5 يومياً | | | | | |
|  | | | | | | |
|  | غير ذلك (نرجو التحديد) | | | | | |
| \|  \| \| --- \| | | | | | | |
|  | | | | | | |
| 1. **ما نوع القهوة التي تشربها عادةً؟** | | | | | | |
|  | | | | | | |
|  | قهوة فورية / قهوة عربية | | | | | |
|  | | | | | | |
|  | نسكافيه | | | | | |
|  | | | | | | |
|  | بدائل القهوة | | | | | |
|  | | | | | | |
|  | لا أشرب القهوة | | | | | |
|  | | | | | | |
|  | غير ذلك (نرجو التحديد) | | | | | |
| \|  \| \| --- \| | | | | | | |
|  | | | | | | |
| 1. **كم فنجان قهوة تشرب يومياً؟** | | | | | | |
|  | | | | | | |
|  | لا أتناول أي فنجان قهوة | | | | | |
|  | | | | | | |
|  | فنجاناً واحداً | | | | | |
|  | | | | | | |
|  | فنجانين | | | | | |
|  | | | | | | |
|  | 3 فناجين | | | | | |
|  | | | | | | |
|  | 4 فناجين | | | | | |
|  | | | | | | |
|  | 5 فناجين | | | | | |
|  | | | | | | |
|  | أكثر من 5 فناجين | | | | | |
|  | | | | | | |
| 1. **هل سمعت سابقاً عن تكوين مركب ضار يتكون عند ارتفاع درجة حرارة بعض الأطعمة التي تحتوي على الكربوهيدرات (مثل الخبز والبطاطا) وتحولها إلى اللون البني؟** | | | | | | |
|  | | | | | | |
|  | نعم | | | | | |
|  | | | | | | |
|  | كلا (انتقل إلى السؤال رقم 34) | | | | | |
|  | | | | | | |
| 1. **ما هي في رأيك الآثار الصحية السلبية لهذا المركب الضار؟** | | | | | | |
|  | | | | | | |
|  | لا أعلم | | | | | |
|  | | | | | | |
|  | أنا أعلم أنً (يرجى التحديد في المربع أدناه) | | | | | |
|  | | | | | | |
|  | قد تكون الاثار الصحيًة سلبية | | | | | |
| \|  \| \| --- \| | | | | | | |
|  | | | | | | |
| 1. **من أي مصدر حصلت على معلوماتك عن هذا المركب الضار؟ (ضع إشارة في المربعات المناسبة).** | | | | | | |
|  | | | | | | |
|  | التلفاز | | | | | |
|  | | | | | | |
|  | الإنترنت/ وسائل التواصل الاجتماعي | | | | | |
|  | | | | | | |
|  | الجريدة/ المجلة | | | | | |
|  | | | | | | |
|  | من شخص (عائلة/أصدقاء) | | | | | |
|  | | | | | | |
|  | المدرسة/ الجامعة | | | | | |
|  | | | | | | |
|  | هيئة سلامة الغذاء في لبنان | | | | | |
|  | | | | | | |
|  | غير ذلك (نرجو التحديد) | | | | | |
|  | | | | | | |
| 1. **"الأكريلاميد مركب ضار يتكون عندما تصبح بعض الأطعمة الكربوهيدراتية بنية اللون، وذلك بسبب ارتفاع درجة الحرارة"، هل يجب أن تحتوي عبوات الطعام على معلومات حول المحتوى والمستوى الآمن من مادة الأكريلاميد (على سبيل المثال الحد الأقصى اليومي)؟** | | | | | | |
|  | | | | | | |
|  | نعم، بالتأكيد | | | | | |
|  | | | | | | |
|  | كلا، بالتأكيد | | | | | |
|  | | | | | | |
|  | ليس لدي رأي راسخ | | | | | |
|  | | | | | | |
| 1. **برأيك، هل يجب أن تحتوي عبوات المواد الغذائية على معلومات حول التكون المحتمل لمادة الأكريلاميد في المنتج إذا لم يتم اتباع تعليمات الطهي بشكل صحيح؟** | | | | | | |
|  | | | | | | |
|  | | نعم، بالتأكيد | | | | |
|  | | | | | | |
|  | | كلا، بالتأكيد | | | | |
|  | | | | | | |
|  | | ليس لدي رأي راسخ | | | | |
|  | | | | | | |
| 1. **برأيك، إذا كان ملصق المنتج يذكر مادة الأكريلاميد بمستوى آمن (أو أدنى من الحد اليومي الموصى به)، فهل تشتري المنتج؟** | | | | | | |
|  | | | | | | |
|  | | نعم، بالتأكيد | | | | |
|  | | | | | | |
|  | | كلا ، بالتأكيد | | | | |
|  | | | | | | |
|  | | غير متأكد | | | | |
